# Structural Differences at Quadruplex‐Duplex Interfaces Enable Ligand‐Induced Topological Transitions

**DOI:** 10.1002/advs.202309891

**Published:** 2024-03-13

**Authors:** Yoanes Maria Vianney, Dorothea Dierks, Klaus Weisz

**Affiliations:** ^1^ Institut für Biochemie Universität Greifswald Felix‐Hausdorff‐Str. 4 D‐17489 Greifswald Germany

**Keywords:** induced fit, intercalation, NMR spectroscopy, Phen‐DC_3_, quadruplex‐duplex junction

## Abstract

Quadruplex‐duplex (QD) junctions, which represent unique structural motifs of both biological and technological significance, have been shown to constitute high‐affinity binding sites for various ligands. A QD hybrid construct based on a human telomeric sequence, which harbors a duplex stem‐loop in place of a short lateral loop, is structurally characterized by NMR. It folds into two major species with a (3+1) hybrid and a chair‐type (2+2) antiparallel quadruplex domain coexisting in a K^+^ buffer solution. The antiparallel species is stabilized by an unusual capping structure involving a thymine and protonated adenine base AH^+^ of the lateral loop facing the hairpin duplex to form a T·AH^+^·G·C quartet with the interfacial G·C base pair at neutral pH. Addition and binding of Phen‐DC_3_ to the QD hybrid mixture by its partial intercalation at corresponding QD junctions leads to a topological transition with exclusive formation of the (3+1) hybrid fold. In agreement with the available experimental data, such an unprecedented discrimination of QD junctions by a ligand can be rationalized following an induced fit mechanism.

## Graphical abstract

A G‐quadruplex featuring a coaxially stacked duplex stem‐loop folds into a major antiparallel topology stabilized by an unusual capping base quartet. Binding of a Phen‐DC_3_ ligand through partial intercalation at the quadruplex‐duplex junction disrupts the capping structure and enforces a topological transition to a hybrid‐type G‐quadruplex fold.

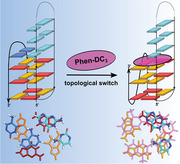

## 1 Introduction

Tetra‐stranded G‐quadruplexes (G4s) have attracted great interest for their presence and regulatory roles in living cells,^[^ ^1^ ^]^ but also for their use in various technological applications, e.g., as aptamers, sensors, and DNAzymes.^[^ ^2^, ^3^ ^]^ A prominent feature of these nucleic acid structures is their remarkable polymorphism. In general, canonical G4s can be grouped into three families depending on relative strand orientations, namely parallel, (3+1) hybrid, and (2+2) antiparallel G4s with four, three, and two out of four strands of the same 5′‐to‐3′ backbone polarity. For monomolecular G4s, the four G‐strands or G‐columns are linked by propeller, lateral, or diagonal loops progressing in a clockwise or counter‐clockwise direction. Being directly related to mutual strand polarities, the arrangement and type of loops are determinants of the various G4 topologies.^[^ ^4^ ^]^

Owing to their biological role and technological exploitation, G4s have been targeted by a plethora of small ligands, e.g., for their visualization in cells,^[^ ^5^ ^]^ for cancer therapeutic strategies,^[^ ^6^ ^]^ or for the detection of analytes.^[^ ^7^ ^]^ Many of the G4‐specific ligands comprise a polycyclic aromatic ring system for maximizing stacking interactions with an outer G‐tetrad.^[^ ^8^ ^]^ High‐affinity G4 binding by such planar ligands has often been successful on parallel G4s, frequently found in oncogenic promoter sequences and thus considered a promising target for novel cancer therapies.^[^ ^9^, ^10^ ^]^ This can be attributed to the lack of lateral and diagonal loops that may hamper free ligand access to 5′‐ or 3′‐terminal G‐tetrads. However, flanking sequences may also have a profound influence on the ligand binding^[^ ^11^, ^12^ ^]^ and given unique tertiary folds when in a longer sequence context as suggested,^[^ ^13^ ^]^ relationships between truncated G4 structures, binding affinity, and biological activity are far from being straightforward in most cases.

Recently, quadruplex‐duplex (QD) junctions have been recognized as high‐affinity binding sites for various ligands, binding at the interface of the G4 and a coaxially stacked duplex domain.^[^ ^14^, ^15^, ^16^, ^17^, ^18^, ^19^ ^]^ Notably, such QD junctions are suggested to frequently occur in vivo and to provide for novel regulatory signals.^[^ ^20^, ^21^, ^22^ ^]^ Additionally, QD hybrids offer to be promising tools for the assembly of expanded DNA architectures, the engineering of specific G4 folds guided through duplex formation, and the design of biosensors or nanocircuits.^[^ ^23^, ^24^, ^25^ ^]^ In the present studies, a QD model system was designed based on a blunt‐ended human telomeric sequence and a first TTA loop substituted with a duplex hairpin‐forming sequence. Using high‐resolution NMR methods, different G4 topologies for two coexisting QD hybrid structures were found and characterized in detail. Remarkably, addition of a Phen‐DC_3_ ligand selected for a single species based on its unprecedented discrimination of the two different high‐affinity QD junctions. Possible mechanisms for shifts in equilibria are discussed based on the experimental findings.

## 2 Results and Discussion

### 2.1 The QD Hybrid Adopts Two Major Coexisting Topologies

**Figure 1:**
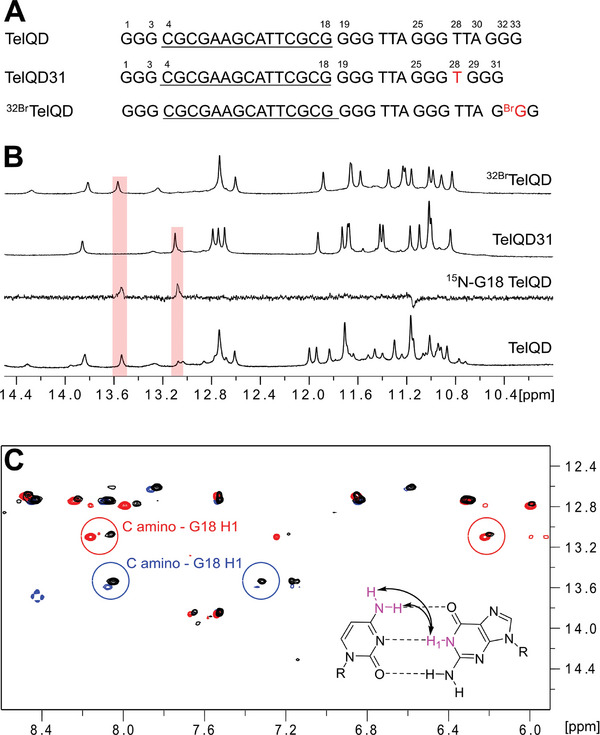
A) Sequence of native TelQD as well as modified TelQD31 and ^32Br^TelQD with some of the residues numbered and modifications highlighted in red; underlined nucleotides mark a putative hairpin‐forming first loop. B) Watson–Crick and Hoogsteen imino proton NMR spectral region of TelQD, TelQD31, and ^32Br^TelQD. Also shown is a ^15^N‐filtered 1D HMQC spectrum using ^15^N‐G18 labeled TelQD (10% ^15^N enrichment). C) Superimposed H8/amino(ω_2_)‐H1(ω_1_) NOESY spectral regions of TelQD (black), TelQD31 (red), and ^32Br^TelQD (blue). Characteristic NOE contacts as expected for G18 H1 and neighboring C amino protons in a GC base pair are circled. Spectra were acquired at 25 °C in a 120 mm K^+^ buffer, pH 7.0.

The design of the QD hybrid termed TelQD makes use of a previously employed self‐complementary oligonucleotide,^[^ ^26^ ^]^ incorporated in the present studies as a first intervening loop region within a human telomeric sequence (**Figure** **1**A). Looking at the imino proton NMR spectral region of TelQD acquired in a 120 mm K^+^ buffer, Hoogsteen imino proton resonances between 10.6 and 12.0 ppm indicate the coexistence of different G4 folds with additional formation of Watson‐Crick base pairs as suggested by more downfield‐shifted imino resonances in the range 12.4–14.4 ppm (Figure 1B).

Employing a site‐specific ^15^N‐enrichment of G18 at the 3′‐terminal position of the first intervening sequence and thus adjacent to the putative G‐core in a folded species, two G18 imino proton signals with chemical shifts >13 ppm could be identified at 25 °C (Figure 1B). These point to two major QD hybrid topologies with G18 H1 engaged in an interfacial GC Watson‐Crick base pair of a lateral duplex stem‐loop, expected to comprise six Watson–Crick GC and AT base pairs capped by a 3‐nt GCA hairpin loop. Note, that formation of propeller but also diagonal loops places 5′‐ and 3′‐terminal bases of the hairpin domain at a distance too far for efficient base pairing.^[^ ^26^, ^27^ ^]^

In an attempt to select for a single species for its structural characterization, two sequence variants were designed. Notably, (3+1) hybrid‐2 and antiparallel G4s constitute typical G4 topologies that allow for the formation of a first lateral duplex stem‐loop. Thus, in modified sequence TelQD31 the last three‐nucleotide (3‐nt) TTA loop was replaced by a 1‐nt T loop whereas in sequence ^32Br^TelQD a *syn*‐favoring 8‐bromo‐dG was incorporated into the central position 32 of the 3′‐terminal G‐run (Figure 1A). Importantly, the two modified sequences showed different but well‐resolved imino proton NMR spectral regions, both in line with a single G4 species (Figure 1B). Also, each G18 imino proton resonance found for the parent sequence was again identified in one of the two modified oligonucleotides. Additional confirmation that TelQD31 and ^32Br^TelQD represent the two major folds of parent TelQD comes from a superposition of corresponding NOESY spectral regions. Thus, typical NOE cross‐peaks between G18 H1 and amino protons of a base‐paired cytosine as observed for TelQD31 and ^32Br^TelQD closely match the pair of G18 H1 – C amino NOE contacts for the native TelQD (Figure 1C).

Testing any potential G4 associations of the hybrid constructs, gel electrophoresis was performed using a three‐tetrad *c‐myc* G4 and a six‐tetrad human telomeric (HT) dimer as a reference. Major bands for all sequences studied here migrated faster than the dimeric HT control, confirming their folding into a monomolecular species (Figure S1, Supporting Information).

### 2.2 TelQD Folds into a (3+1) Hybrid and a Chair‐Type (2+2) Antiparallel Topology

Shortening of the last 3‐nt into a 1‐nt loop for modified TelQD31 will strongly favor appropriate propeller loop formation but bears the risk of promoting global folding into an all*‐anti* parallel G4 structure.^[^ ^28^ ^]^ However, the observation of five strong intra‐nucleotide H8‐H1’ NOE cross‐peaks typical of *syn*‐residues essentially excludes a parallel fold and can be attributed to the energetic benefit of additional hydrogen bond and stacking interactions when forming a first lateral duplex stem‐loop.^[^ ^24^ ^]^ In the following, NMR spectral assignments were performed using well‐established strategies for the analysis of several homonuclear and heteronuclear 2D NMR experiments and were further confirmed by site‐selectively ^15^N‐enriched oligonucleotides (Figures S2–S4, Supporting Information). In addition to the identification of five *syn‐*guanosines located in a three‐layered G‐core, uninterrupted sequential NOE walks from G1 to G18 point to a first lateral loop coaxially stacked above the G‐tetrad plane (Figure S2, Supporting Information).

**Figure 2:**
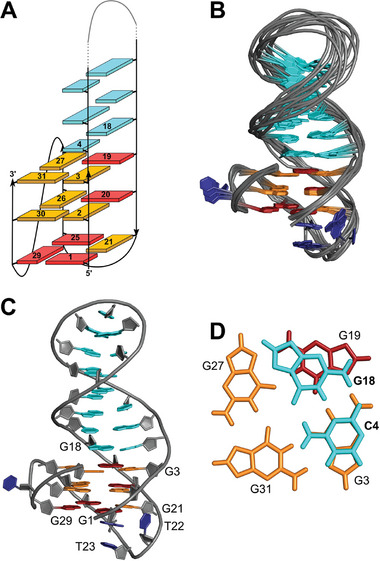
Structure of TelQD31. A) Schematic structural representation. B) Superposition of 10 lowest‐energy structures; G10‐C11‐A12 loop bases of the duplex hairpin are omitted for clarity. C) Representative structure of TelQD31. D) Top view of the QD interface with the interfacial C4·G18 Watson–Crick base pair stacked onto the 3′‐outer G‐tetrad. *Anti*‐ and *syn‐*guanosines are colored in orange and red, respectively; residues in the lateral hairpin loop are shown in cyan whereas residues of the two other loops are colored dark blue.

Structure calculations with NMR restraints for TelQD31 resulted in a (3+1) hybrid‐2 structure characterized by a ‐(llp) topology according to the notation introduced by Webba da Silva (**Figure** **2**A, for structural statistics see Table S3, Supporting Information).^[^ ^4^ ^]^ By progressing in a counter‐clockwise direction, the first extended duplex stem‐loop bridges a wide groove, matching in groove dimensions with the duplex minor groove to allow for a smooth transition from the G‐core to the duplex domain (Figure 2). The interfacial C4·G18 base pair coaxially stacks above G3 and G19 of the 3′‐outer tetrad (Figure 2D). T22 of the well‐converged second TTA loop is located in the wide groove whereas T23 is stacked below A24 that caps the 5′‐tetrad below G21 (Figure 2C). Such an arrangement bears resemblance to a 3‐nt loop with a py‐py‐pu motif running across a narrow groove and lacking any additional tertiary interactions from other loop or overhang domains.^[^ ^24^, ^28^ ^]^

For ^32Br^TelQD with an 8‐bromo‐dG substitution, five strong H8‐H1’ cross‐peaks suggest the presence of six *syn‐*guanosines when including the *syn*‐favoring G analog lacking a corresponding cross‐peak. Such equal numbers of *syn*‐ and *anti*‐Gs within the G‐core already point to the formation of a (2+2) antiparallel G‐quadruplex with three tetrad layers. Again, NMR resonances were fully assigned employing standard strategies based on homo‐ and heteronuclear 2D NMR experiments and were further supported by site‐selectively ^15^N‐enriched oligonucleotides (Figures S5–S8, Supporting Information). The detailed analysis confirmed the presence of six *syn‐*guanosines, namely, G1, G19, G20, G25, G31, and ^Br^G32, all of them participating in G‐tetrad formation. In addition, continuous NOE contacts from G1 to G18 again suggest formation of a duplex domain coaxially stacked onto a terminal G‐tetrad plane.

Notably, chemical shifts of both H8 and H1 of G18 in the interfacial Watson‐Crick base pair are downfield‐shifted in comparison to corresponding protons of other G residues within the duplex domain. Also, upon lowering the temperature the presence of another most downfield‐shifted signal at ≈14.3 ppm becomes clearly apparent (Figures S9A and S10, Supporting Information). This resonance was unambiguously identified to be H1 of protonated AH^+^30 through site‐specific ^15^N isotope labeling (Figure S9B, Supporting Information). In addition, an upfield shift of almost 10 ppm, well established as a marker for adenine protonation, was observed for its ^13^C2 in a ^1^H‐^13^C HSQC spectrum (Figure S11, Supporting Information).^[^ ^29^ ^]^ Although participation of protonated adenine bases with elevated p*K*_a_ values to form base pairs, base triads, or base quartets has been reported for RNA and DNA structures in the past,^[^ ^30^, ^31^, ^32^, ^33^, ^34^ ^]^ observation of a corresponding AH^+^ imino proton at neutral pH is remarkable and suggests a unique local environment with a strong stabilization of the protonated adenine base.

**Figure 3:**
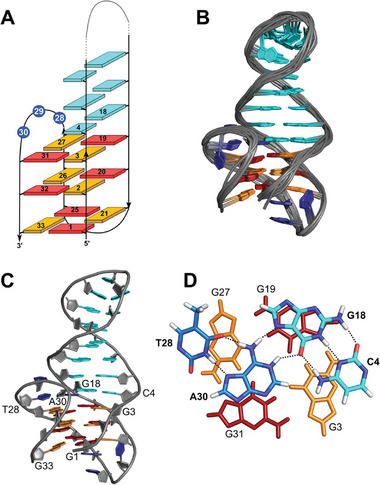
Structure of ^32Br^TelQD. A) Schematic structural representation. B) Superposition of 10 lowest‐energy structures. C) Representative structure. D) Top view of the formed quartet C4·G18·A30·T28 capping the 3′‐outer G‐tetrad and comprising a protonated adenine. *Anti*‐ and *syn‐*guanosines are colored in orange and red, respectively; residues in the lateral hairpin loop are shown in cyan whereas residues of the two other loops are colored dark blue.

Restrained molecular dynamics calculations yielded structural details of the ^32Br^TelQD fold. The topology is found to be a chair‐type (2+2) antiparallel G4 with three lateral loops running in a counter‐clockwise direction (**Figure** **3**). Generally, antiparallel folds are considered to be disfavored in a K^+^ solution. However, antiparallel G4 structures comprising both homopolar and heteropolar tetrad stackings have previously been reported in a potassium buffer.^[^ ^35^, ^36^ ^]^ The present structure closely resembles the chair‐type fold of a human telomeric sequence with a single A‐to‐T substitution to allow for an inter‐loop AT interaction.^[^ ^35^ ^]^ Adopting the same *syn*‐*anti* pattern with a first *syn*‐*anti*‐*anti*‐G‐column of the G‐core, the latter only differs from ^32Br^TelQD by the clockwise progression of its lateral loops in a +(lll) topology. Clearly, it is the coaxially stacked duplex stem‐loop with its strong propensity to bridge a G4 wide groove for a smooth G4‐to‐duplex transition that enforces a counter‐clockwise –(lll) loop progression in the ^32Br^TelQD hybrid.

Interestingly, a hydrogen bond‐mediated base quartet capping the 3′‐outer tetrad is formed by the interfacial CG base pair and A30 as well as T28 located within the lateral loop facing the duplex hairpin (Figure 3D). Remarkably, A30 is protonated at pH 7 and hydrogen‐bonded to the Hoogsteen face of guanine in the CG base pair as firmly established by experimental observations. As seen in all calculated structures, there is an additional, albeit more dynamic, binding of loop residue T28 to the Hoogsteen face of protonated AH^+^30, keeping the latter in place for optimal interactions with the CG base pair. Formation of such a base quartet bears close resemblance to a quartet reported for a GTP‐binding RNA aptamer.^[^ ^32^ ^]^ In the latter case, a protonated adenine of an internal bulge binds to a CG Watson‐Crick base pair of a central helix in full analogy to the QD hybrid but with a 2‐nt preceding G within the bulge instead of a T further hydrogen‐bonded to AH^+^.

Additional loop‐helix interactions to form a base quartet stacked on the 3′‐outer tetrad are expected to stabilize a chair topology even in a K^+^‐containing buffer. This is also reflected in mutational studies (Figure S12, Supporting Information). Thus, replacing A30 for a thymine base or changing the interfacial CG into a GC base pair inevitably disrupts the antiparallel fold of ^32Br^TelQD. On the other hand, substituting T28 or T29 by a cytosine base had less dramatic or essentially no effects, respectively. These observations are in line with only weaker additional T28·AH^+^30 interactions, yet the imino proton spectral region of the 28T>C mutant showed increased heterogeneity together with an upfield shift and decreasing intensity of the AH^+^30 imino resonance.

### 2.3 Binding of Phen‐DC_3_ Induces Refolding of the Antiparallel into a (3+1) Hybrid Topology

**Figure 4:**
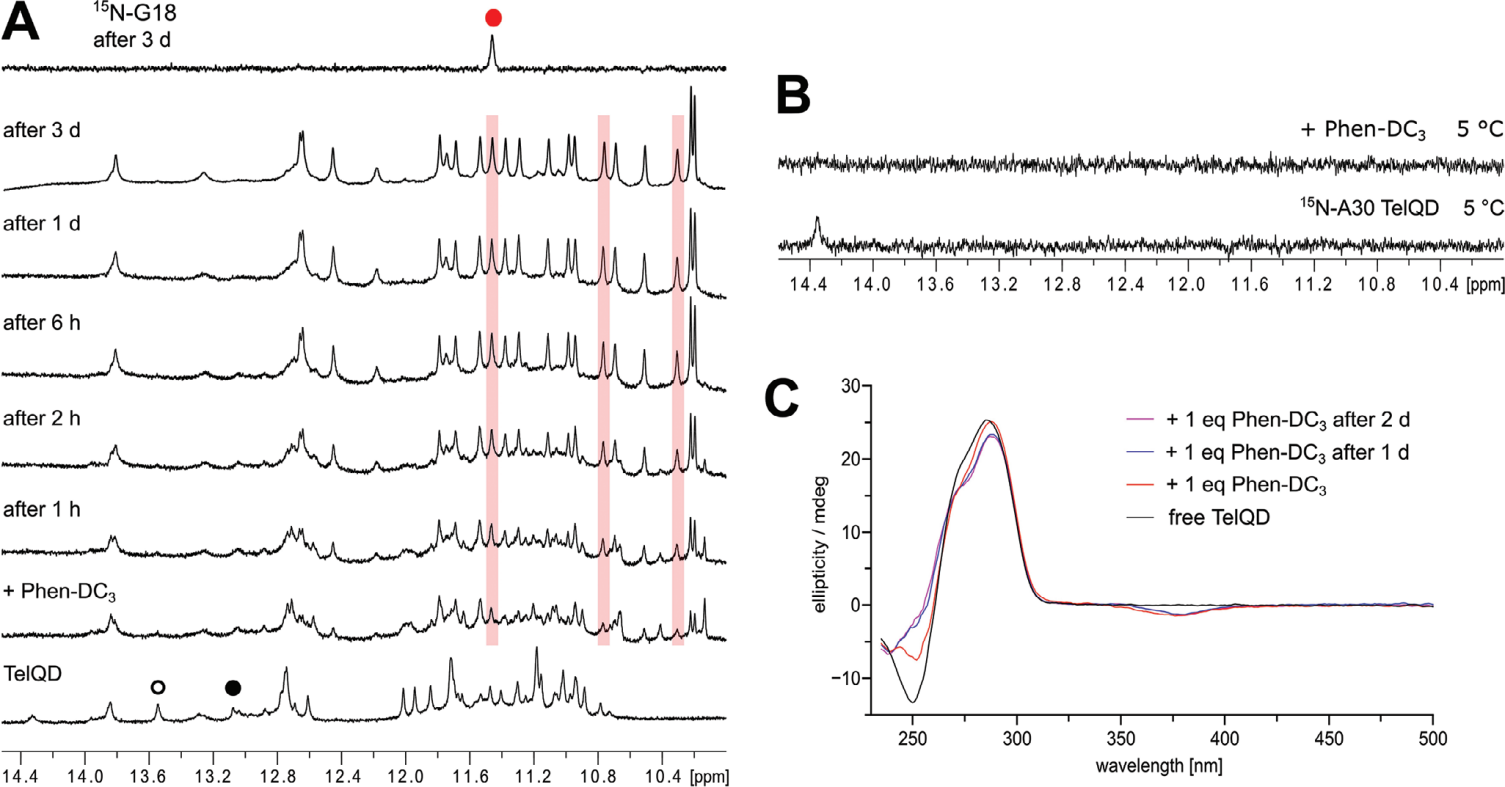
A) Imino proton NMR spectral region of TelQD acquired before (bottom) and at different time intervals after addition of 1 eq. Phen‐DC_3_ at 25 °C. Shown at the top is a corresponding ^15^N‐filtered 1D HMQC spectrum of a ^15^N‐G18 labeled TelQD sequence (10% ^15^N enrichment) acquired after 3 days following ligand addition. The build‐up of an upfield‐shifted G18 imino resonance of the complex, marked by the red circle, is traced together with two other imino signals by vertical red lines. G18 imino resonances of coexisting antiparallel and hybrid‐2 topologies of free TelQD as identified through corresponding ^15^N‐editing experiments are marked by a black open and solid circle, respectively. B) ^15^N‐edited imino proton spectral region of a ^15^N‐A30 labeled TelQD sequence (10% ^15^N enrichment) acquired before (bottom) and directly after addition of 1 eq. Phen‐DC_3_ at 5 °C. C) CD spectra of free and Phen‐DC_3_ bound TelQD recorded immediately or 1–2 days after ligand addition at 25 °C. All samples were dissolved in a 120 mm K^+^ buffer, pH 7.0.

Immediately following addition of one equivalent Phen‐DC_3_ to TelQD, the imino proton spectral region shows significant polymorphism with several more upfield‐shifted resonances also including ligand protons. However, after six hours of incubation it changes to a nicely resolved one‐component spectrum with only small admixtures of very minor species (**Figure** **4**A). Apparently, the imino resonance of AH^+^30 vanishes immediately after ligand addition even at lower temperatures used to promote sharper signals (Figure 4B). Whereas AH^+^30 and G18 imino resonances of the free antiparallel hybrid seem to instantly disappear upon ligand addition, a G18 imino proton of a newly formed complex gradually grows in intensity during a longer time period as do other imino resonances. This imino proton is significantly upfield‐shifted when compared to G18 H1 of free TelQD. Likewise, deviating from typical (3+1) hybrid and antiparallel signatures through superimposed CD effects also including the duplex domain, the CD spectral shape of TelQD changes with time (Figure 4C). The minimum and maximum amplitudes at 250 and 285 nm show a continuous decrease associated with small bathochromic shifts within one day after ligand addition. In contrast, fast binding of the ligand is indicated by a negative induced CD effect in the ligand long‐wavelength absorption region at 375 nm having fully developed directly following Phen‐DC_3_ addition.

**Figure 5:**
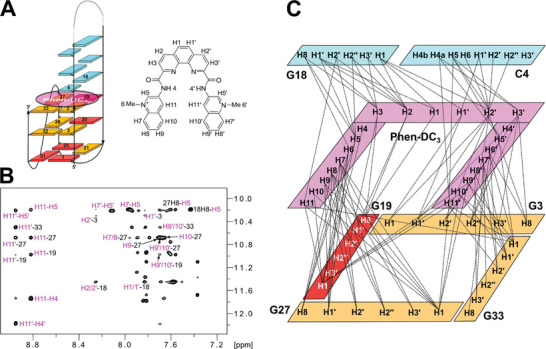
A) Chemical structure of Phen‐DC_3_ with atom numbering and schematic representation of the 1:1 TelQD – Phen‐DC_3_ complex; *anti*‐ and *syn*‐G residues of the G‐core are colored orange and red whereas base pairs of the duplex stem‐loop and the ligand are colored cyan and magenta, respectively. B) Portion of a 2D NOESY spectrum showing intra‐ and intermolecular Phen‐DC_3_ cross‐peaks; G H1 protons are labeled in black with residue numbers while ligand protons are labeled in magenta. NOESY spectra were acquired with a 300 ms mixing time at 25 °C in a 120 mm K^+^ buffer, pH 7.0. C) Schematic representation of observed intermolecular NOE contacts between Phen‐DC_3_ and TelQD protons at the QD interface.

A more detailed structural analysis of the formed complex through NOESY, DQF‐COSY, and ^1^H‐^13^C HSQC experiments allowed for the identification of five G residues adopting a *syn* glycosidic conformation. Also, NOE walks show high similarities with the hybrid‐2 topology of TelQD31 (for resonance assignment strategies see the Supporting Information and Figures S13–S17, Supporting Information). However, there is a discontinuity of the sequential walk at the QD interface. On the other hand, various phenanthroline protons of the ligand show NOE contacts to protons located in both the G‐tetrad and the duplex base pair at the junction. For the two ligand quinoline sidearms, NOE connectivities are not only observed to protons directly positioned at the QD junction but also to protons located at the exposed side of the interfacial G‐tetrad (**Figure** **5**). A total of 100 intermolecular ligand‐DNA NOE contacts fix the Phen‐DC_3_ alignment and demonstrate its capping of the 3′‐outer G‐tetrad in a partial intercalative binding mode.

Structure calculations employing NMR restraints showed good convergence to yield a structural ensemble consistent with the NMR data (for structural statistics see Table S9, Supporting Information). Larger distance restraint violations were found for the TTA propeller and the GCA loop of the duplex hairpin. This can be attributed to their greater flexibility, but especially for the propeller loop violations may also result from an inadequate force field and its shortcomings for fixing the residues during the simulation.^[^ ^37^ ^]^

**Figure 6:**
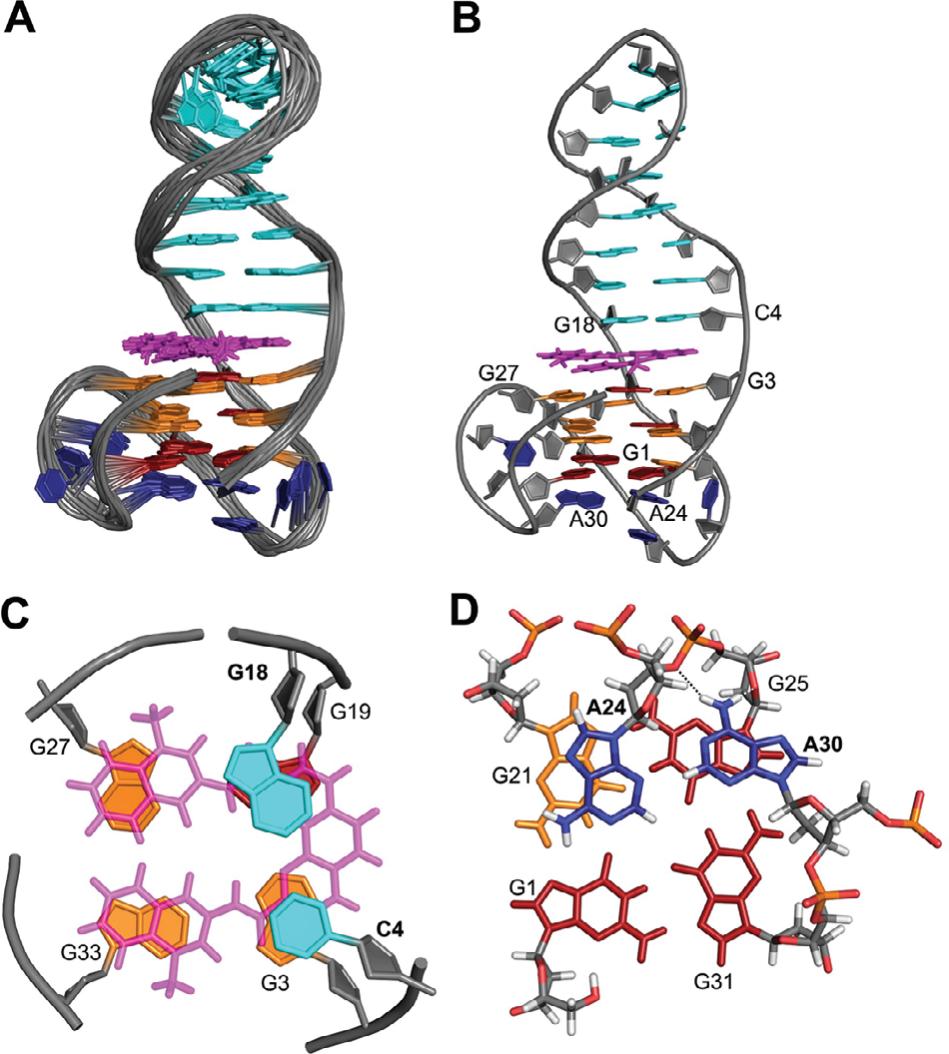
Structure of TelQD complexed with Phen‐DC_3_. A) Superposition of ten lowest‐energy structures. B) Representative structure. C) View onto the QD interface with Phen‐DC_3_ intercalated at the junction. D) View onto A24 and A30 capping the 5′‐outer tetrad. *Anti*‐ and *syn‐*guanosines are colored in orange and red whereas residues within the duplex hairpin loop and in the other two loops are colored in cyan and dark blue, respectively; Phen‐DC_3_ is colored in magenta.

The Phen‐DC_3_ ligand binds through intercalation of its phenanthroline tricyclic ring system between the C4·G18 base pair and G3 and G19 of the 3′‐tetrad at the QD interface. The two quinoline sidearms cover the exposed side of the tetrad, capping G27 and G33 bases (**Figure** **6**). Such an arrangement with partial intercalation at the junction maximizes the *π−π* overlap between the four guanines of the terminal tetrad and the quinoline and phenanthroline moieties of the ligand and is reminiscent of a recently reported complex between Phen‐DC_3_ and a parallel QD hybrid.^[^ ^14^ ^]^ It is also responsible for the high‐affinity binding at the QD junction and for the G4 selectivity of Phen‐DC_3_, strongly disfavoring double‐helical DNA. Thus, Phen‐DC_3_ is expected to show inferior stacking upon duplex intercalation with its two quinoline groups protruding into solvent to also impose a significant entropic penalty.^[^ ^8^ ^]^

The 5′‐outer tetrad is capped by A24 of the second TTA lateral loop as also observed for the free hybrid‐2 structure formed by TelQD31. However, enabled by the longer 3‐nt propeller loop in native TelQD, terminal A30 of the latter loop also participates in forming an A24·A30 capping structure stacked onto G21 and G25 of the tetrad. This arrangement, supported by corresponding NOE cross‐peaks, seems to be a more common feature and has been observed in other G4s comprising corresponding loop domains.^[^ ^28^, ^38^ ^]^ In the present structure, the A30 residue seems to be additionally fixed by putative hydrogen bonds formed between its amino protons and A24 O3’ and G25 O4’ of the sugar‐phosphate backbone (Figure 6D).

### 2.4 Ligand‐Mediated Refolding: Conformational Selection versus Induced Fit

Time‐dependent spectral changes of the native TelQD hybrid after Phen‐DC_3_ addition reveal two processes proceeding with different rates. Whereas initial ligand binding is fast, there is also a slow structural rearrangement from a coexisting major (2+2) antiparallel to a (3+1) hybrid‐2 topology. In line with these observations, ligand titration to TelQD31 that exclusively folds into a hybrid‐2 structure immediately yields a single well‐defined 1:1 complex (Figure S18A, Supporting Information). A more detailed NMR spectral analysis of the formed TelQD31 – Phen‐DC_3_ associate confirms Phen‐DC_3_ binding at the QD junction of the conserved G4 structure in close analogy to the corresponding TelQD complex (for resonance assignment strategies see the Supporting Information and Figures S19–S22, Supporting Information).

Likewise, ligand addition to antiparallel chair‐type ^32Br^TelQD results in the immediate disappearance of resonances and the appearance of new upfield‐shifted imino signals (Figure S18B, Supporting Information). Thus, the most downfield‐shifted AH^+^30 and the well‐resolved G18 imino resonance of the free QD hybrid are almost completely lost directly after ligand addition and small remaining signals possibly due to the DNA being in slight excess do not further change with time. In agreement with fast initial binding, ligand addition is also associated with the immediate emergence of a fully developed negative induced CD effect of bound ligand at ≈375 nm (Figure S18C, Supporting Information). On the other hand, ongoing changes for other imino resonances, suggesting some structural rearrangements, are apparent for up to 6 h after ligand addition. However, the ligand fails to induce complete refolding of ^32Br^TelQD into a (3+1) hybrid topology and there is significant polymorphism even after days of equilibration in contrast to unmodified TelQD. Clearly, the incorporation of a *syn*‐favoring 8‐bromo‐dG at position 32 in ^32Br^TelQD is expected to stabilize the antiparallel conformer due to its matching positioning while destabilizing a hybrid‐2 fold with conformational mismatch at the bromo‐dG insertion site, resulting in a shift of the corresponding equilibrium.

**Figure 7:**
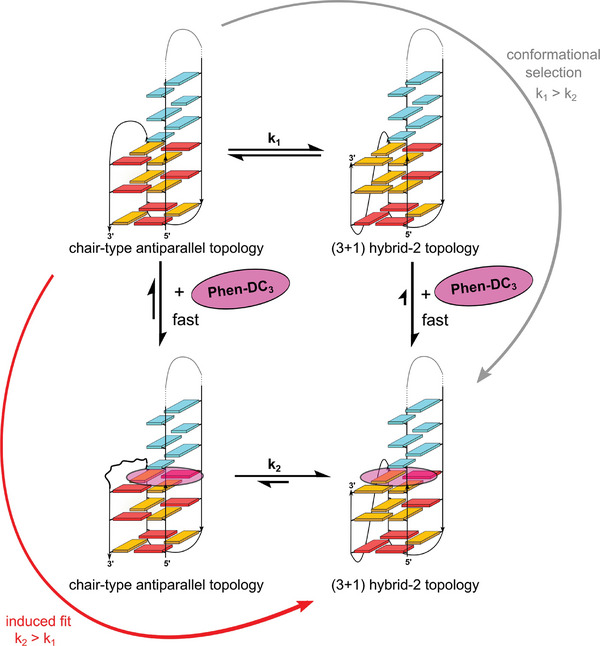
Simplified thermodynamic cycle for the Phen‐DC_3_‐mediated refolding through conformational selection or induced fit. Transitions between the two QD hybrids characterized by rate constants k_1_ and k_2_ may involve various intermediates. Note that the structure for the ligand‐bound antiparallel QD hybrid features a disrupted lateral loop alignment but could not be characterized in detail (bottom left).

Previously, the natural product epiberberine was shown to induce extensive structural rearrangements upon its binding to a hybrid‐2 telomeric G4 and experimental observations suggested its ability to at least partially convert other telomeric G4 topologies into the hybrid‐2 form.^[^ ^39^ ^]^ In an additional in‐depth structural study on a ligand‐induced G4 refolding, Phen‐DC_3_ shifted the topology of a telomeric sequence from a hybrid‐1 to an antiparallel chair‐type conformation with the formation of a complex with Phen‐DC_3_ intercalated between tetrads.^[^ ^40^ ^]^ Compatible with the difficulty of direct ligand intercalation between G‐tetrads, the ligand was suggested to bind pre‐folded short‐lived intermediates based on a conformational selection mechanism to guide folding to the final complex. In contrast to the latter report, binding of Phen‐DC_3_ at a QD interface in the present studies shifts the equilibrium toward an exclusive (3+1) hybrid structure. Here, the interconversion may take different pathways as shown in the simplified cycle of **Figure** **7**. Whereas ligand binding is fast, topological transitions expected to proceed through various intermediates are associated with partial unfolding and are reflected in the slow kinetics of interconversions seen in time‐dependent NMR experiments. Consequently, relative rates of antiparallel to (3+1) hybrid transitions k_1_ and k_2_ will effectively determine the predominant pathway (Figure 6). For k_1_ > k_2_, the process follows a conformational selection. For k_2_ > k_1_, the mechanism is rather characterized by an induced fit model.

Experimental results presented here demonstrate that a capping quartet involving the third TTA lateral loop stabilizes the antiparallel chair‐type topology. Blocking free access to the exposed part of the outer tetrad at the QD junction, ligand binding disrupts such a structure. As a consequence, there will be an energetic penalty upon Phen‐DC_3_ binding but a subsequent lowering of the transition free energy when interconverting into a (3+1) hybrid topology. Such a refolding can be rationalized by an energy‐consuming dissociation of the fourth G‐column of the antiparallel conformer to form a triplex intermediate followed by strand reorientation of the fourth G‐tract into an inverted alignment with formation of a propeller loop. Of note, folding pathways of telomeric sequences have also been suggested to proceed through pre‐folded triplex structures.^[^ ^41^ ^]^ Therefore, given a fast ligand binding at the QD junction associated with disruption of the chair‐stabilizing capping structure that involves residues of the lateral loop, k_2_ > k_1_ can be anticipated. Thus, being compatible with experimental observations the topological transition is suggested to follow an induced fit mechanism.

## 3 Conclusion

Hairpin‐forming intervening sequences of a G‐rich domain were shown to drive G4 formation into specific topologies able to accommodate a favorable coaxial stacking of a lateral duplex stem‐loop onto an outer G‐tetrad. Additional tertiary interactions of the interfacial base pair with lateral loop or flanking residues may easily guide folding of the G4 into topologies usually considered disfavored under corresponding conditions. Thus, a human telomeric sequence comprising a first lateral duplex stem‐loop folds into two major coexisting species: A (3+1) hybrid‐2 conformer with a second lateral and a final propeller loop as well as a (2+2) chair‐type structure with a second and third lateral loop stabilized by additional loop‐base pair interactions. In contrast to the hybrid‐2 structure, the latter is expected to impose steric hindrance to high‐affinity binding of a Phen‐DC_3_ ligand, characterized by its partial intercalation at the QD junction together with ligand sidearms covering the full surface area of the G‐tetrad. Remarkably, the ligand enforces refolding of the chair‐type antiparallel topology into the hybrid‐2 topology through its discriminating binding at the QD interface. Based on a fast disruption of the chair‐stabilizing loop interaction promoted by Phen‐DC_3_ stacking on the outer G‐tetrad, an induced fit mechanism may likely be operative for the conversion to a more stable hybrid‐2 complex.

In showing the impact of QD junctions on G4 folding, the present studies are expected to support the prediction of folds for G‐rich natural sequences comprising putative QD interfaces. Importantly, they also highlight new design principles for developing G4 architectures for different applications. On the other hand, ligand‐induced refolding of the G4 domain in a QD hybrid demonstrates the complex interplay of interactions. Whereas topological conversions guided by the binding of ligands or proteins may set limits to a rational targeting of such interconverting G4 structures in vivo, they also offer additional possibilities in the design of molecular switches driven by specific ligands. Alternatively, pH changes will affect protonation of the adenine base, thus stabilizing or disrupting the capping structure to possibly also enable pH‐driven structural transitions. Finally, the unprecedented discrimination of different QD junctions as high‐affinity binding hotspots for various ligands may hold great promise for a more selective high‐affinity targeting of G4 structures in the future.

## Conflict of Interest

The authors declare no conflict of interest.

## Supporting information

Supporting Information

## Data Availability

Atomic coordinates and chemical shifts for TelQD31 (PDB ID 8R6D, BMRB ID 34880), ^32Br^TelQD (PDB ID 8R6G, BMRB ID 34881) and for TelQD with bound Phen‐DC_3_ (PDB ID 8R6H, BMRB ID 34882) have been deposited in the Protein Data Bank and in the Biological Magnetic Resonance Bank.
