# Supplementary material for: Structural Differences at Quadruplex‐Duplex Interfaces Enable Ligand‐Induced Topological Transitions
Source: Adv Sci (Weinh). 2024 Mar 13;11(24):2309891. doi: 10.1002/advs.202309891 (PMC11200018; doi:10.1002/advs.202309891)
Supplement: Supplementary file 1 — Supporting Information [file ADVS-11-2309891-s001.pdf]

ADVANCED SCIENCE
Open Access

Supporting Information

for *Adv. Sci.*, DOI 10.1002/advs.202309891

Structural Differences at Quadruplex-Duplex Interfaces Enable Ligand-Induced Topological Transitions

*Yoanes Maria Vianney, Dorothea Dierks and Klaus Weisz**

Supporting Information

Structural Differences at Quadruplex-Duplex Interfaces Enable Ligand-Induced Topological Transitions

*Yoanes Maria Vianney, Dorothea Dierks, and Klaus Weisz**

Table of Contents

Materials and methods 4

Table S1. Determination of molar extinction coefficient for Phen-DC_3 7

Figure S1. Non-denaturing gel electrophoresis of QD hybrids 7

Strategy for resonance assignments of TelQD31 8

Figure S2. Topology and NOESY spectral regions of TelQD31 10

Figure S3. Guanine imino and H8 proton assignments for TelQD31 11

Figure S4. H2'/H2" assignments for TelQD31 12

Table S2. NMR chemical shifts for TelQD31 13

Table S3. NMR restraints and structural statistics of TelQD31 14

Strategy for resonance assignments of ^{32Br}TelQD 15

Figure S5. H6/H8-H1'/H5 NOESY and DQF-COSY spectral region of ^{32Br}TelQD 17

Figure S6. Topology and NOESY spectral regions of ^{32Br}TelQD 18

Figure S7. Imino and H8 proton assignments for ^{32Br}TelQD 19

Figure S8. H2'/H2" assignments for ^{32Br}TelQD 20

Figure S9. Assignment of the AH^+30 imino proton in ^{32Br}TelQD 21

Figure S10. NOESY spectral regions of ^{32Br}TelQD at 5 °C 22

Figure S11. NOESY and ^1H-^{13}C HSQC spectral regions of ^{32Br}TelQD at 5 °C 23

Figure S12. Imino proton spectral region of ^{32Br}TelQD mutants 24

Table S4. NMR chemical shifts for ^{32Br}TelQD 25

Table S5. NMR restraints and structural statistics of ^{32Br}TelQD 26

Strategy for resonance assignments of the TelQD – Phen-DC_3 complex 27

Figure S13. Imino and H8 proton assignments for the TelQD – Phen-DC_3 complex 29

Figure S14. NOESY and COSY spectral regions of the TelQD – Phen-DC_3 complex 30

Figure S15. NOESY spectral regions of the TelQD – Phen-DC_3 complex 31

Figure S16. ^{1}H-^{13}C HSQC spectral regions of the TelQD – Phen-DC_3 complex 32

Figure S17. H2'/H2" assignments for the TelQD – Phen-DC_3 complex 33

Table S6. NMR chemical shifts for TelQD complexed with Phen-DC_3 34

Table S7. NMR chemical shifts for Phen-DC_3 in a complex with TelQD 35

Table S8. Intermoleculat Phen-DC_3-TelQD NOE contacts 36

Table S9. NMR restraints and structural statistics of a TelQD – Phen-DC_3 complex 38

Figure S18. Time-dependent NMR and CD spectra following ligand addition 39

Strategy for resonance assignments of the TelQD31 – Phen-DC_3 complex 40

Figure S19. NOESY spectral regions of the TelQD31 – Phen-DC_3 complex 41

Figure S20. Imino and H8 proton assignments for the TelQD31 – Phen-DC_3 complex 42

Figure S21. ^{1}H-^{13}C HSQC spectral regions of the TelQD31 – Phen-DC_3 complex 43

Figure S22. H1 and H8 chemical shift perturbations upon TelQD31 complexation 43

Table S10. NMR chemical shifts for TelQD31 complexed with Phen-DC_3 44

References 45

Materials and methods

Materials and sample preparation

^{15}N- and $^{15}N,^{13}C$-labeled 2'-deoxyribonucleoside phosphoramidites were purchased from Eurisotop (Saarbrücken, Germany) and Silantes (München, Germany). DNA oligonucleotides were synthesized by TIBMOLBIOL (Berlin, Germany) and further purified by ethanol precipitation. Concentrations were determined by absorbances A_{260} at 80 ºC in water using molar extinction coefficients as provided by the manufacturer. Before measurements, all samples were annealed by heating to 90 ºC for 5 min followed by slow cooling to room temperature. Two buffer systems were used: A low-salt buffer with 10 mM potassium phosphate, pH 7.0, and a high-salt buffer with 20 mM potassium phosphate, pH 7.0, supplemented with 100 mM KCl.

Phen-DC_3 was purchased from Sigma-Aldrich (Taufkirchen, Germany). The concentration of Phen-DC_3 was determined by its absorbance using a molar extinction coefficient ε_{351} = 27.327 $M^{-1}\cdot cm^{-1}$, initially established at 25 ºC in DMSO (see Table S1). A concentrated Phen-DC_3 stock solution in DMSO was prepared for ligand titration.

Circular dichroism (CD)

CD spectra were recorded with a Jasco J-810 spectropolarimeter equipped with a Peltier thermostat. Phen-DC_3 was titrated to the QD hybrid constructs (5 µM) in a low- or high-salt buffer as stated in the text. Spectra were acquired with a bandwidth of 1 nm, a scanning speed of 50 $nm\cdot min^{-1}$, a response time of 4 s, a data pitch of 1 nm, and five accumulations. All spectra were blank-corrected.

Non-denaturing gel electrophoresis

Oligonucleotides with a strand concentration of 100 µM were dissolved in a high-salt buffer. For complexes, Phen-DC_3 was added and the solution incubated at room temperature for 5 days to ensure equilibration. Samples were mixed with an equal volume of a glycerol-buffer mixture (40% $^{v}/_{v}$). Oligonucleotides (500 pmol per lane) were loaded and separated by electrophoresis in TBE buffer, pH 8.3, supplemented with 120 mM KCl at 10 ºC overnight. The gel was stained with thiazole orange (5 µM) and the bands were visualized under UV light.

NMR spectroscopy

NMR spectra were acquired with a Bruker Avance Neo 600 MHz spectrometer equipped with an inverse $^{1}H/^{13}C/^{15}N/^{19}F$ quadruple resonance cryoprobehead and z-field gradients. Data were processed in Topspin 4.0.7 and analyzed in CcpNmr V2.[1] Proton chemical shifts were referenced indirectly to sodium trimethylsilylpropionate (TSP) following the temperature-dependent water chemical shift at pH 7. ^{13}C and ^{15}N chemical shifts were referenced relative to sodium trimethylsilylpropanesulfonate (DSS) and to liquid ammonia, respectively.

For the NMR experiments, samples (0.2-1 mM) in either a low-salt or high-salt buffer system with 90% H_2O/10% D_2O or 100% D_2O were used. 1D ^{1}H NMR spectra were acquired with a relaxation delay of 2 s, employing a WATERGATE with w5 element for solvent suppression, and were subsequently processed with an exponential window function. For 2D NMR experiments with a 90% H_2O/10% D_2O solvent, water suppression techniques differed depending on the particular experiment. NOESY spectra were recorded with 80, 150, and 300 ms mixing times, a WATERGATE-w5 pulse sequence for solvent suppression, and 2K x 1K data points. ^{1}H-^{13}C HSQC and DQF-COSY experiments were performed with a 3-9-19 pulse scheme and 4K x 500 data points whereas ^{1}H-^{13}C HMBC spectra were acquired with a jump-and-return pulse sequence, a delay $\Delta_{1/2J}$ of 21 ms, and 2K x 60 data points. In case of a 100% D_2O buffer system, water presaturation was used in DQF-COSY and NOESY experiments (80 and 300 ms mixing times).

Samples with 10% isotope enrichment were used for site-selectively ^{15}N- and ^{13}C-labeled oligonucleotides. 1D ^{15}N-edited HMQC spectra were acquired using a selective excitation pulse for the imino proton (Q5.1000 scheme with a 90º flip angle) followed by a standard HMQC pulse scheme with ^{15}N decoupling during acquisition. The delay time was optimized for a coupling constant J_{NH} of 90 Hz. For the detection of $^{15}N7$-H8 correlations, a modified HMQC pulse sequence published previously was used.[2] A Q3.1000 pulse scheme with a 180º flip angle and a bandwidth of ~40 ppm for the ^{15}N channel serves to selectively refocus N9-H8 scalar couplings. An additional 90º purge pulse was employed before acquisition and the delay optimized for a coupling constant of 16 Hz. A 2D ^{1}H-^{15}N HMQC spectrum on a site-selectively ^{15}N-labeled oligonucleotide was acquired with a jump-and-return water suppression scheme and 4K x 256 data points.

NMR restraints and structure calculations

Distance restraints were grouped based on 2D NOE cross-peak intensities. For non-exchangeable protons, distances were set to 2.9 ± 1.1 Å for very strong cross-peaks, 4.0 ± 1.2 Å for strong cross-peaks, 5.5 ± 1.5 Å for weak cross-peaks, and 6.0 ± 1.5 Å for very weak cross-peaks. For exchangeable protons, distances for weak and very weak cross-peaks were set to 5.0 ± 1.2 Å and 6.0 ± 1.2 Å, respectively. Distance restraints involving AH^+ H1 and ligand protons were set to 2.9 ± 1.1 Å for very strong cross-peaks, 4.0 ± 1.5 Å for strong cross-peaks, 5.0 ± 1.5 Å for weak cross-peaks, and 6.0 ± 1.5 Å for very weak cross-peaks. Restraints for ambiguous cross-peaks were set to 5.0 ± 2.0 Å. Glycosidic torsion angles χ were restrained to either *anti* (170º-310º) or *syn* (25º-95º). All evaluated deoxyribose sugar puckers were restrained to south with a pseudorotational phase angle of 144º-180º. Additional hydrogen bond and planarity restraints were added for the G-tetrads and the duplex base pairs. Chirality restraints were imposed on all residues.

Initially, distance geometry simulated annealing was used to generate 100 starting structures with XPLOR-NIH 3.0.3.[3] Structural refinement was done in AMBER18 with the parmbsc force field and OL15 modifications through simulated annealing with a generalized Born solvent model.[4] Restraints were applied as given above but with planarity restraints for the duplex base pairs released. Parametrization for 8-bromo-2'-deoxyguanosine, N1-protonated adenosine, and Phen-DC_3 was done using the R.E.D server.[5] Geometry optimization and partial atomic charges were calculated with the Hartree-Fock method and a 6-31G* basis set. Force field parameters were adapted from parm10 and GAFF. For refinement in explicit TIP3P water,[6] two potassium ions were added in the center between two G-tetrads. The system was further neutralized with K^+ ions, only imposing NMR-based and hydrogen bond distance restraints. The simulation and employed force constants followed the protocol as described recently.[7] After 4 ns of simulation, the trajectory was averaged for the last 500 ps and further minimized in implicit solvent to give ten lowest-energy structures. RMSD values were calculated with the VMD program[8] and structures visualized with the PYMOL molecular graphics system.[9]

Table S1. Determination of the Phen-DC3 molar extinction coefficient ε_{351} by concentration-dependent absorbances A_{351}.[a]

conc.	10.5 µM	15.0 µM	19.5 µM	24.0 µM	28.5 µM	ε_{351} ($M^{-1}\cdot cm^{-1}$)[b]	B[b]
A_{351}	0.2755	0.4033	0.5318	0.6522	0.7760	27669.2	0.012
	0.2890	0.4182	0.5267	0.6640	0.7892	27682.0	-0.0026
	0.2964	0.4199	0.5300	0.6560	0.7775	26628.8	0.017
ε_{351} = 27327 ± 604 $M^{-1}\cdot cm^{-1}$							

[a]Absorbances were determined for three independent concentration series in 1-cm quartz cuvettes at 25 ºC in 100% DMSO. [b]A least-square fit of the data with a linear equation $A = \varepsilon \cdot c + B$ yielded the extinction coefficient ε and the intercept B. Average and standard deviation of ε is given at the last line for the Phen-DC3 absorption maximum at 351 nm.

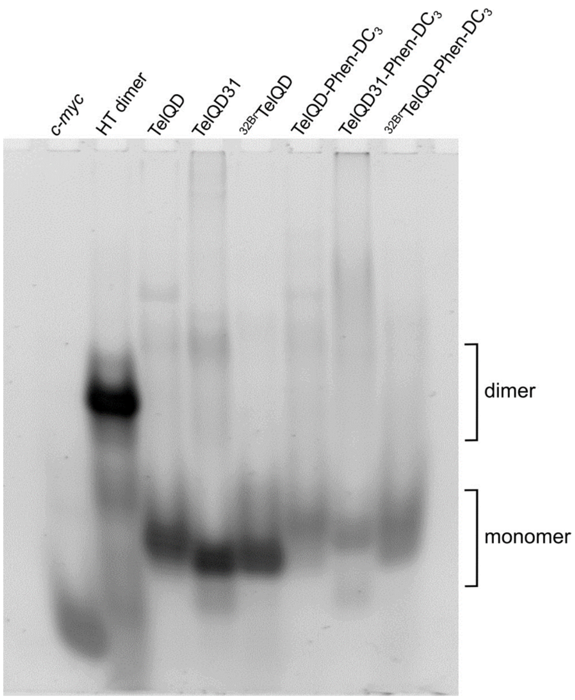

Figure S1. Non-denaturing gel electrophoresis of QD hybrids without and with addition of one equivalent of Phen-DC3. The three-layered *c-myc* G-quadruplex d(TGAGGGTGGGTAGGGTGGGTAA) and the six-layered HT dimer $d(TTAGGG)_8TT$ were used as markers. Migration bands observed for the QD hybrids suggest their major folding into a monomolecular G4.

Spectral assignment of TelQD31

The 1D imino proton spectral region shows twelve peaks for Hoogsteen-bound G imino protons between 10.8 and 12.0 ppm and a total of six Watson-Crick-type imino proton signals between 12.6 and 14.0 ppm. These already suggest formation of a three-tetrad G-quadruplex with a duplex stem-loop comprising six base pairs. Continuous base-sugar NOE connectivities could be followed from G1 to G18, establishing the first G-column as well as the first loop forming a duplex hairpin. Assignments of duplex resonances are in close analogy to a QD hybrid previously assigned by Phan et al..[10] Additional non-interrupted connectivities could be traced from G19 to A24, establishing the second G-column and a second loop (Figure S2). The third and fourth G-column could be differentiated following proton-proton contacts of deoxyribose sugars of G27 and T28 (spectral region not shown). Based on the intensity of intra-residual H8-H1' contacts and supported by the chemical shifts of guanine C8-H8 correlations in a ^{1}H-^{13}C HSQC spectrum (Figure S3E), five G residues were found to be in a *syn* glycosidic conformation. Notably, four rectangular NOE cross-peak patterns between base and sugar protons typical for *syn-anti* steps could be observed for the steps G1 to G2, G20 to G21, G25 to G26, and G29 to G30 (Figure S2C and D). Also, another non-regular NOE cross-peak pattern strongly suggests the presence of a *syn-syn* step between G19 and G20.

Assignments of G H8 and imino protons within a G-tetrad were confirmed through several site-specifically ^{15}N- or ^{15}N/^{13}C-labeled oligonucleotides (Figure S3A-C). Additionally, imino resonances were identified through a ^{1}H-^{13}C HMBC experiment, clearly correlating seven guanine H8 and H1 resonances through their long-range coupling to ^{13}C5 (Figure S3D). From intra-tetrad H8-H1 NOE contacts, tetrad polarities were firmly established. Thus, hydrogen bonds when proceeding from donor to acceptor run along G1→G29→G25→G21 for the bottom tetrad, G2→G20→G26→G30 for the central tetrad, and G3→G19→G27→G31 for the upper tetrad (Figure S2E). As a result, the G-quadruplex domain features both homopolar and heteropolar tetrad stacking, also demonstrated by the absence of continuous NOE connectivities between H8-H1 contacts of the bottom and central tetrad of opposite polarity. Tetrad stacking was further corroborated by imino-imino NOE contacts (Figure S2B). Thus, heteropolar tetrad stacking was demonstrated by strong inter-tetrad H1-H1 cross-peaks observed between G1 and G30 or between G29 and G26. Such an alignment requires first, second, and third intervening sequences to proceed through a lateral, lateral, and propeller loop, respectively. Here, the first lateral loop constitutes a duplex hairpin that bridges the G-quadruplex wide groove.

Duplex imino proton assignments were based on typical NOE contacts to cytosine amino protons and to adenine H2 protons. Notably, sequential and long-range contacts from C4 and G18 to various protons of G3 and G19 were observed, suggesting that the first Watson-Crick base pair C4·G18 is coaxially stacked above the upper tetrad (Figure S2). In general, the G-quadruplex is proposed to fold into a hybrid-2-type topology with a QD junction located at its upper 3'-outer G-tetrad.

Finally, a stereospecific assignment of H2'/H2" protons was achieved through different intensities of the H1'-H2' and H1'-H2" cross-peaks in NOESY spectra acquired with short mixing times. Following assignments, the deoxyribose sugar pucker was evaluated by relative intensities of H1'-H2' and H1'-H2" cross-peaks in a DQF-COSY experiment (Figure S4). Due to their antiphase pattern for active couplings giving rise to cancellation effects, stronger COSY cross-peaks suggest larger coupling constants. All 26 residues that were unambiguously assigned were found to adopt a south-type sugar pucker.

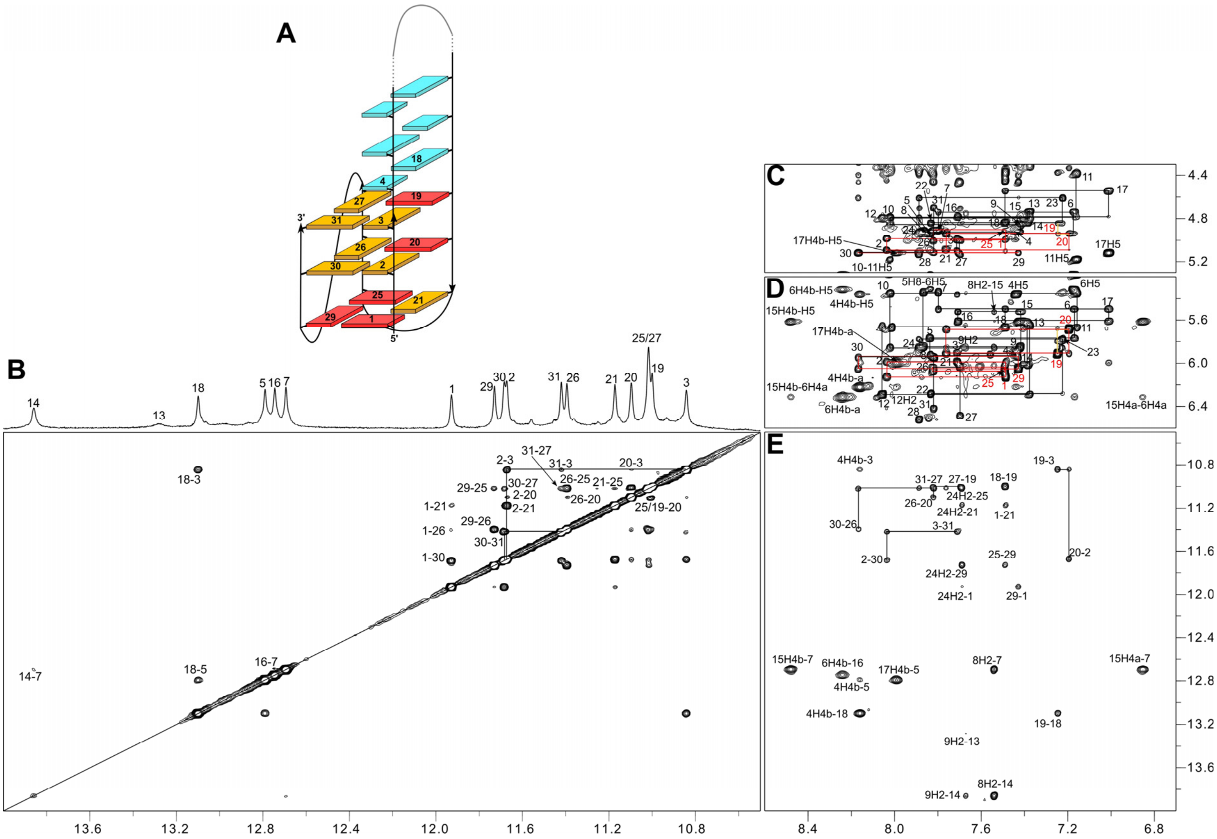

Figure S2. (A) Topology of the TelQD31 hybrid; *anti*- and *syn*-G residues of the G-core are colored orange and red, respectively; base pairs of the duplex stem-loop are colored cyan. (B) H1-H1 2D NOE spectral region and a corresponding 1D spectrum with imino resonance assignments shown on top. (C-E) 2D NOESY spectral regions showing (C) H8/H6(ω_2)-H3'(ω_1), (D) H8/H6(ω_2)-H1'(ω_1), and (E) H8/H6(ω_2)-H1(ω_1) cross-peaks. In C and D, red lines forming a typical rectangular pattern connect two residues in a *syn-anti* step while yellow-colored lines connect the two residues 19 and 20 in a *syn-syn* step; *syn*-residues are labeled in red color. NOESY spectra (300 ms mixing time) were acquired at 25 °C in 20 mM potassium phosphate buffer, pH 7.0, supplemented with 100 mM KCl.

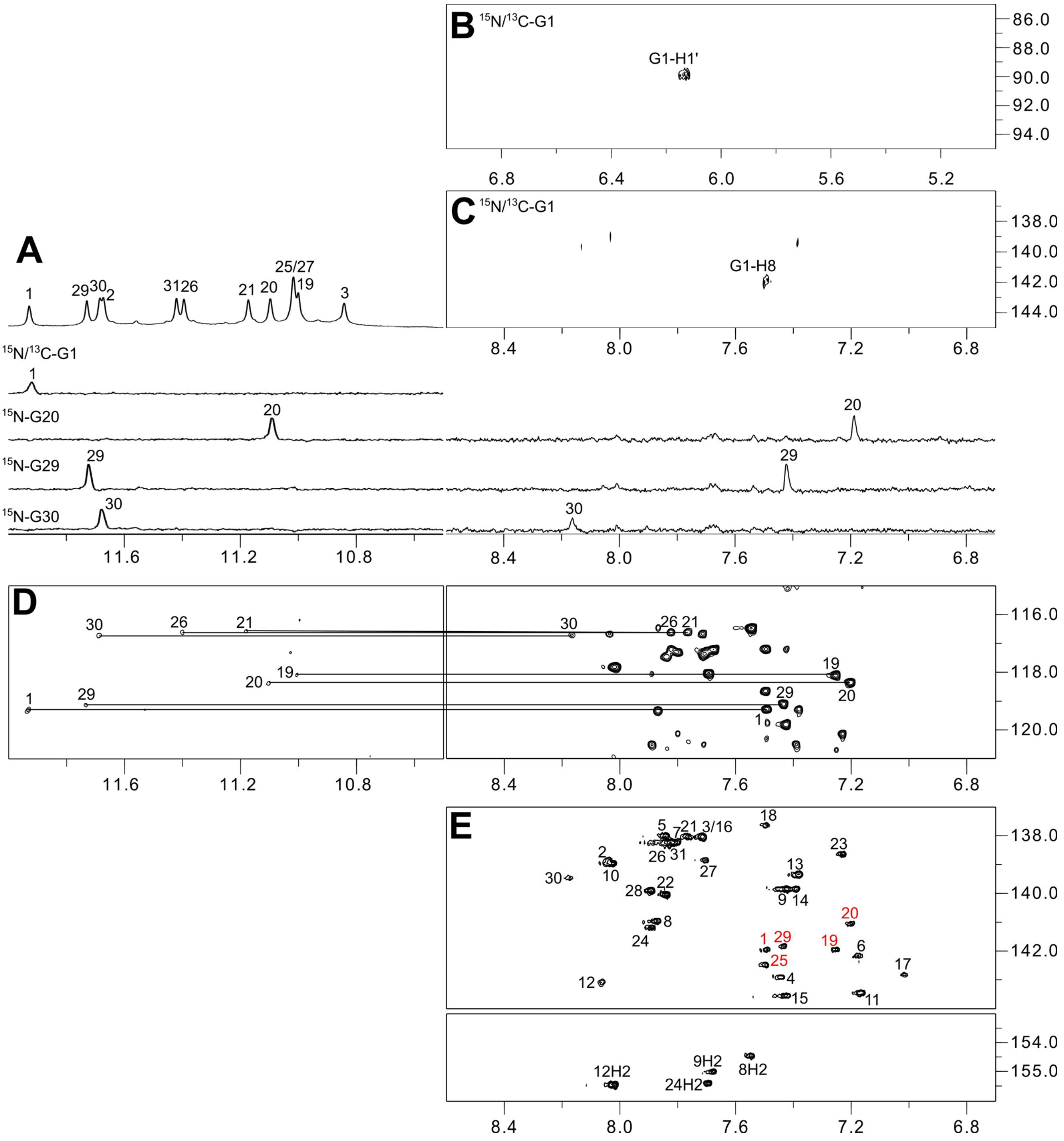

Figure S3. (A) Hoogsteen imino proton spectral region of non-labeled TelQD31 (top) and ^{15}N-edited spectra for TelQD31 sequences site-specifically labeled at G1, G20, G29, and G30 (10% ^{15}N enrichment); except for $^{15}N/^{13}C$ doubly labeled G1, the ^{15}N-edited H8 spectral region is shown to the right. (B,C) Portions of a ^{1}H-^{13}C HSQC experiment on TelQD31 $^{15}N/^{13}C$ labeled at G1 (10% isotope enrichment) showing (B) H1'(ω_2)-C1'(ω_1) and (C) H8(ω_2)-C8(ω_1) correlations. (D) ^{1}H-^{13}C HMBC spectrum of TelQD31 with horizontal lines tracing intra-base G H1(ω_2)-G H8(ω_2) correlations through long-range couplings to $^{13}C5$(ω_1). (E) ^{1}H-^{13}C HSQC spectrum of TelQD31 showing H8/H6-C8/C6 (top) and adenine H2(ω_2)-C2(ω_1) correlations (bottom); *syn*-G residues are labeled with red color.

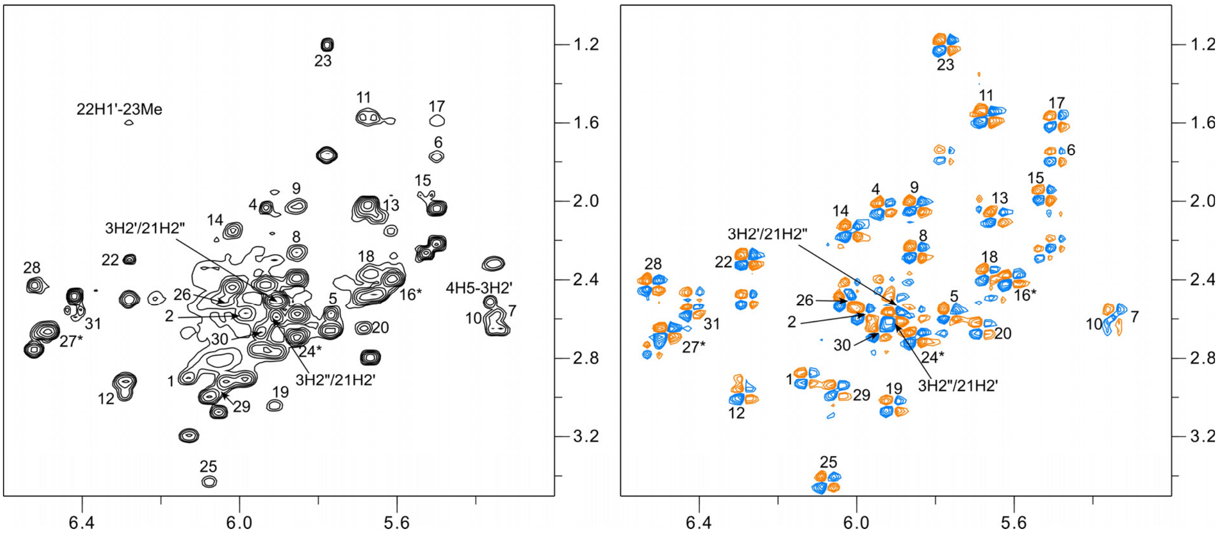

Figure S4. H1'(ω_2)-H2'/H2"(ω_1) spectral region of a NOESY spectrum (left, 80 ms mixing time) and of a DQF-COSY spectrum (right) for TelQD31. Spectra allow a stereospecific assignment of H2'/H2" protons and a sugar pucker evaluation through their cross-peak intensities. Only H1'-H2' cross-peaks are labeled; stars indicate isochronous H2'/H2" resonances.

Table S2. ^{1}H and ^{13}C chemical shifts δ of TelQD31.[a]

δ / ppm	H8/H6	H1/H3	H1'	H2'/H2"	H3'	H5/H2/Me	C8/C6	C2
G1	7.49	11.93	6.13	2.90/3.20	4.99	-	141.95	-
G2	8.04	11.67	5.99	2.58/2.91	5.10	-	138.91	-
G3	7.71	10.85	5.91	2.51/2.60	4.99	-	138.06	-
C4	7.44	-	5.93	2.03/2.43	4.92	5.36	142.91	-
G5	7.84	12.79	5.77	2.58/2.66	4.94	-	138.02	-
C6	7.17	-	5.50	1.77/2.22	4.74	5.32	142.15	-
G7	7.80	12.70	5.34	2.57/2.65	4.91	-	138.25	-
A8	7.87	-	5.85	2.26/2.58	4.92	7.54	140.96	154.47
A9	7.42	-	5.85	2.03/2.39	4.85	7.67	139.86	155.02
G10	8.02	n.d.[b]	5.35	2.60/2.32	4.79	-	138.97	-
C11	7.16	-	5.67	1.57/2.02	4.39	5.18	143.45	-
A12	8.06	-	6.29	2.98/2.92	4.79	8.02	143.13	155.47
T13	7.38	13.28	5.65	2.08/2.48	4.74	1.81	139.35	-
T14	7.39	13.86	6.02	2.15/2.44	4.84	1.59	139.85	-
C15	7.42	-	5.52	1.97/2.26	4.78	5.62	143.56	-
G16	7.71	12.75	5.61	2.40/2.40	4.78	-	138.05	-
C17	7.01	-	5.50	1.59/2.04	4.55	5.12	142.83	-
G18	7.49	13.10	5.67	2.37/2.80	4.84	-	137.64	-
G19	7.25	11.00	5.91	3.04/2.77	4.84	-	141.95	-
G20	7.20	11.10	5.68	2.65/2.49	4.94	-	141.08	-
G21	7.76	11.17	5.91	2.59/2.51	5.09	-	138.03	-
T22	7.83	n.d.[b]	6.28	2.30/2.50	4.84	1.96	140.06	-
T23	7.22	n.d.[b]	5.78	1.20/1.77	4.61	1.60	138.63	-
A24	7.89	-	5.85	2.69/2.69	4.93	7.69	141.18	155.41
G25	7.49	11.02	6.08	3.43/3.00	4.91	-	142.48	-
G26	7.82	11.40	6.03	2.51/2.95	5.00	-	138.25	-
G27	7.70	11.02	6.49	2.67/2.67	5.13	-	138.85	-
T28	7.89	n.d.[b]	6.52	2.43/2.76	5.13	1.99	139.92	-
G29	7.43	11.73	6.05	2.96/3.08	5.12	-	141.82	-
G30	8.17	11.69	5.95	2.67/2.75	5.12	-	139.49	-
G31	7.82	11.42	6.42	2.56/2.48	4.70	-	138.25	-

[a]At 25 ºC in 20 mM potassium phosphate buffer (90% H_2O/10% D_2O), pH 7, supplemented with 100 mM KCl.
[b]n.d. = not determined.

Table S3. NMR restraints and structural statistics of TelQD31.

structure	TelQD31
NOE distance restraints	
intra-residual	136
inter-residual	253
exchangeable	107
other restraints:	
hydrogen bonds	80
dihedral angles	57
planarity	9
chirality	155
structural statistics:	
pairwise heavy atom RMSD value (Å)	
all residues	1.62 ± 0.36
G-tetrad core	0.63 ± 0.14
NOE violations:	
number of NOE violations > 0.2 Å	0
maximum violation (Å)	0.121
mean NOE violation (Å)	0.001 ± 0.0004
deviations from idealized geometry:	
bond lengths (Å)	0.01 ± 0.0001
bond angles (degree)	2.3 ± 0.03

Spectral assignment of ^{32Br}TelQD

The incorporation of an 8-Br-dG analog at position 32 was found to select one species from the two coexisting major folds of native TelQD, allowing for its detailed structural characterization. Having identified cytosine H5-H6 NOE cross-peaks through their corresponding correlations in a DQF-COSY experiment, five strong intra-nucleotide G H1'-H8 NOE cross-peaks typical of *syn*-guanosine residues were observed in a NOESY spectrum acquired with a short mixing time (Figure S5). Thus, with the addition of the *syn*-favoring ^{Br}G32 lacking the H8 proton, the folded ^{32Br}TelQD structure is expected to comprise six *syn*-G residues.

Observation of non-interrupted NOE contacts from G1 to G18 in a NOESY spectrum with a 300 ms mixing time identified the first G-column (Figure S6C,D). The second G-column was largely assigned based on sequences with site-specifically ^{15}N-enriched G19, G20, and G21 nucleotides (Figure S7A). A continuous NOE walk could be traced from G21 to T24. Finally, the third G25-G26-G27 and the fourth G31-G32-G33 G-columns were unambiguously distinguished through ^{32Br}TelQD sequences with selective isotope labeling (Figure S7). Also, sequential NOE connectivities from G27 to T28, T29, and finally A30 established the third G-column. Watson-Crick hydrogen-bonded imino protons of the duplex domain were assigned through their strong NOE contacts between guanine imino and cytosine amino protons as well as between thymine imino and adenine H2 protons (Figure S6E). Conspicuously, the rather broad H8 and Watson-Crick hydrogen-bonded imino proton of G18, expected to border on the outer G-tetrad, are significantly downfield-shifted. From typical NOE patterns associated with *syn-anti* and *syn-syn* steps, the first and the third G-column could be found to comprise *syn-anti-anti* steps whereas the second G-column follows a *syn-syn-anti* arrangement. Consequently, with a non-propeller third loop, the fourth G-column must likewise comprise *syn-syn-anti* steps in line with the brominated ^{Br}G32 favoring a *syn* conformation.

Complete assignments of G H1 and H8 resonances are based on sequential NOE connectivities and supported by ^{15}N-edited spectra of site-specifically ^{15}N-enriched oligonucleotides. An additional D_2O-to-H_2O exchange experiment confirmed and complemented previous assignments. Changing the solvent from 100% D_2O to 90% H_2O, four G imino resonances including G2, G20, and G26 remained unobservable following a 20-minute delay time. These identified residues participating in the solvent-protected central tetrad of the quadruplex (Figure S7B). Consequently, the residual imino proton of centrally located 8-brominated ^{Br}G32 was easily assigned through a process of elimination. Tetrad polarities were

established based on intra-tetrad H1-H8 cross-peaks and additionally supported by H1-H1 inter-tetrad NOE contacts (Figure S6B,E). Following the direction from hydrogen bond donor to acceptor the G-tetrads are aligned according to G1→G33→G25→G21, G2→G20→G26→G32, and G3→G19→G27→G31. Notably, there is a mix of homopolar and heteropolar tetrad stacking and a topology that only comprises lateral loops proceeding in a counterclockwise direction to fold into a chair-type (2+2) antiparallel quadruplex. Finally, 29 out of 33 residues were unambiguously found to adopt a south-type sugar pucker based on the H1'-H2'/H2" cross-peak pattern in a DQF-COSY experiment (Figure S8).

NOE contacts from A30 H2 to C4 H5 as well as to C4 and C17 amino protons suggest that the A30 residue faces the lateral duplex stem-loop (Figure S6D). On the other hand, a closer look on the imino proton spectral region revealed another, yet unassigned very broad and deshielded imino signal at about 14.3 ppm that shows a NOE contact to A30 H2 (Figure S6B,E). Upon decreasing the temperature to 5 °C, this resonance sharpened to now show NOE contacts to G3 and G31 imino protons in the upper tetrad and also to various protons of the interfacial CG and the following GC base pair of the hairpin domain (Figure S10). These indicate a hydrogen-bonded imino proton participating in a capping structure in-plane with the C4·G18 base pair of the duplex stem-loop. Conspicuously, ^{13}C2 of loop adenine A30 was found to be upfield-shifted by ~10 ppm compared to typical ^{13}C2 chemical shifts of the other adenine bases in a ^{1}H-^{13}C HSQC spectrum (Figure S11B). Such an upfield shift was shown to be a reliable marker of adenine N1 protonation and assignment of this most downfield-shifted proton to protonated AH^{+}30 at pH 7 was additionally confirmed by its specific ^{15}N labeling (Figure S9B). Having demonstrated full conservation of the QD structure (Figure S9A), it should be noted that the latter experiments at 5 °C have been performed in a low-salt buffer with 10 mM K^{+} to further increase the spectral signal-to-noise ratio. These conditions also allowed the unambiguous confirmation of the A30 H2 assignment based on an intra-nucleotide H2-H1' NOE contact (Figure S11A).

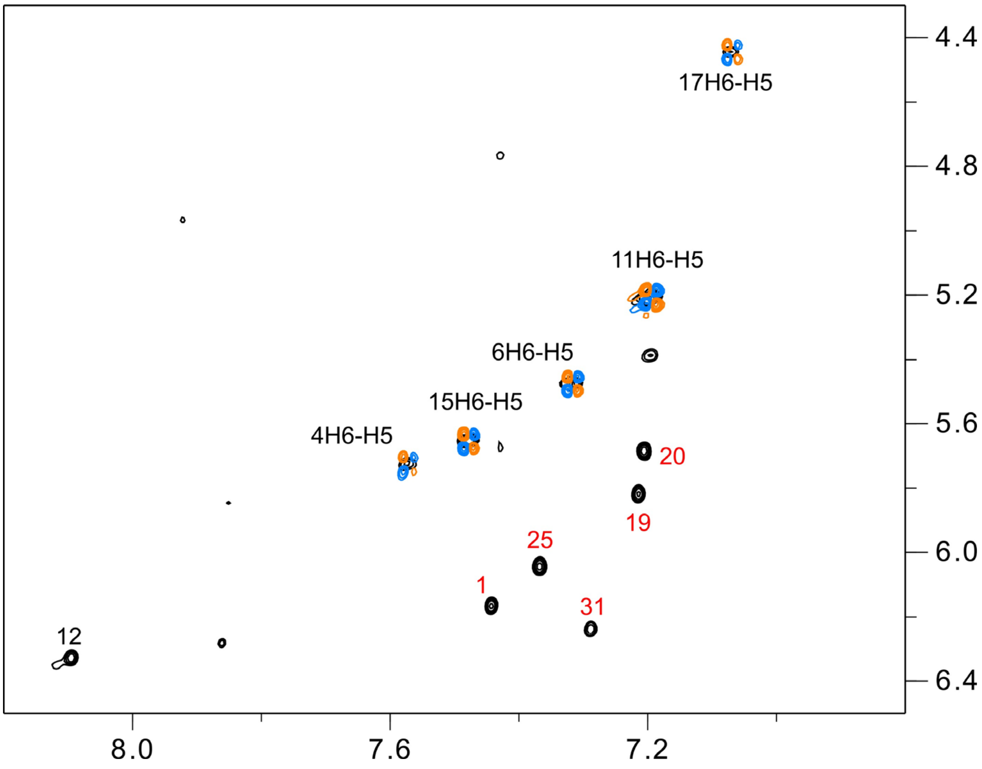

Figure S5. Superposition of the H6/H8(ω_2)-H1'/H5(ω_1) spectral region in a NOESY spectrum (80 ms mixing time, black) and in a DQF-COSY spectrum (orange-blue colored antiphase peaks) of ^{32Br}TelQD. In addition to assigned A12, five other cross-peaks labeled in red have a conspicuously strong intensity (shown for an elevated threshold level), suggesting a *syn* glycosidic conformation. Cytosine H6(ω_2)-H5(ω_1) contacts are discriminated through their COSY cross-peaks. Spectra were acquired at 20 °C in a 20 mM potassium phosphate buffer (100% D_2O), pH 7, supplemented with 100 mM KCl.

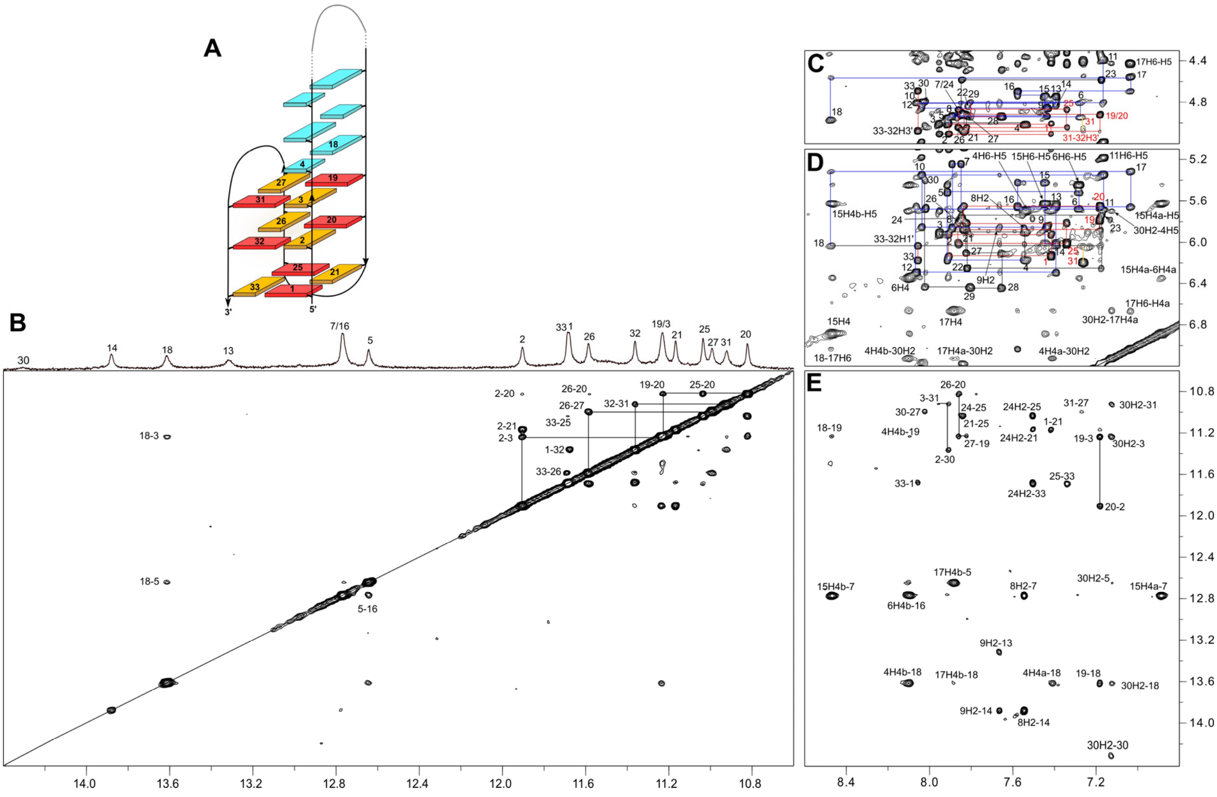

Figure S6. (A) Topology of the ^{32Br}TelQD hybrid; *anti*- and *syn*-G residues of the G-core are colored orange and red, respectively; base pairs of the duplex stem-loop are colored cyan. (B) H1-H1 2D NOE spectral region and a corresponding 1D spectrum with imino resonance assignments shown on top. (C-E) 2D NOESY spectral regions showing (C) H8/H6(ω2)-H3'(ω1), (D) H8/H6(ω2)-H1'(ω1), and (E) H8/H6(ω2)-H1(ω1) cross-peaks. In C and D, red lines forming a typical rectangular pattern connect two residues in a *syn-anti* step while yellow-colored lines connect two residues in a *syn-syn* step; *syn*-residues are labeled in red color. NOESY spectra (300 ms mixing time) were acquired at 20 °C in 20 mM potassium phosphate buffer, pH 7.0, supplemented with 100 mM KCl.

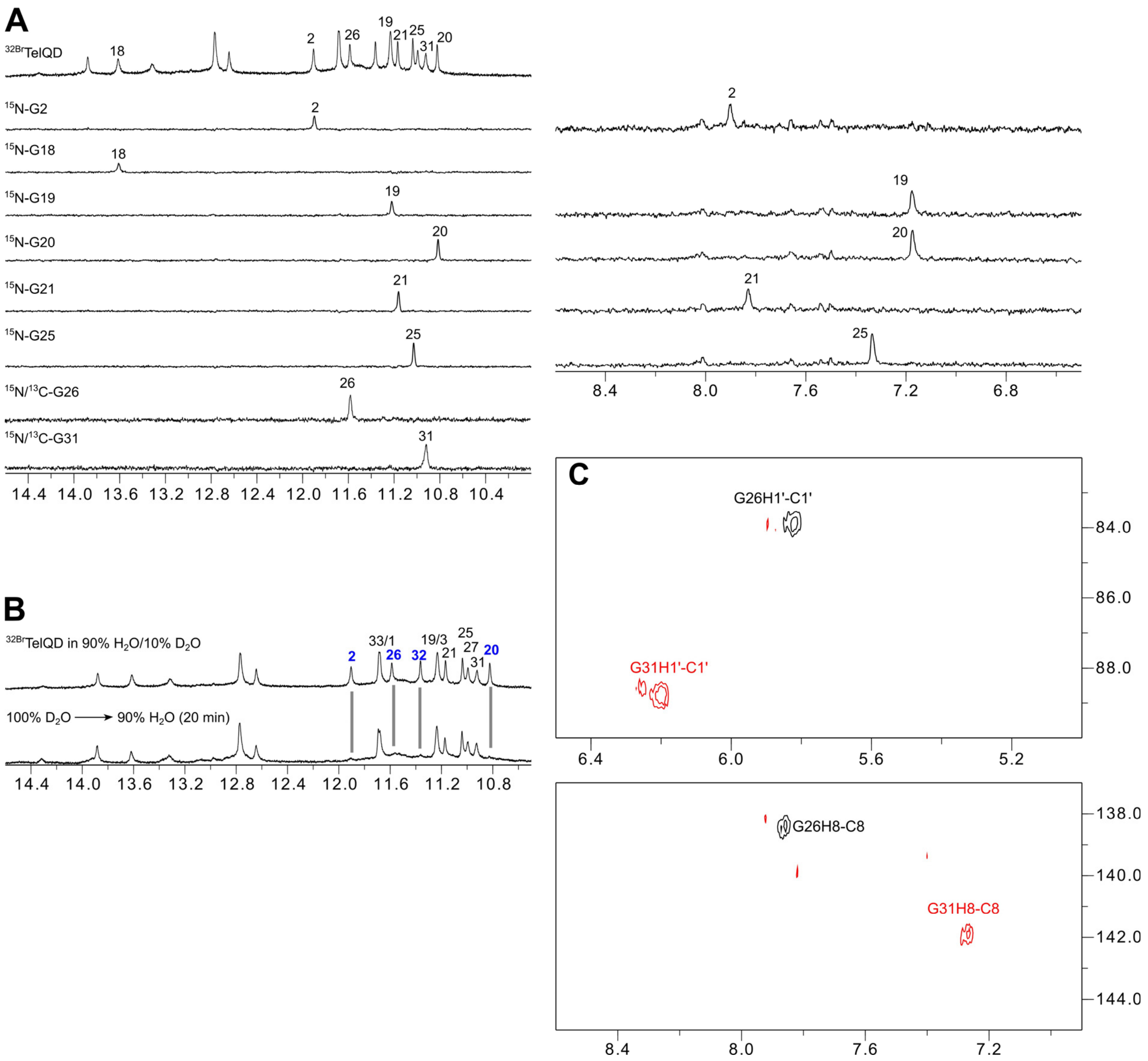

Figure S7. (A) Imino proton spectral region of non-labeled ^{32Br}TelQD (top) and ^{15}N-edited spectra for ^{32Br}TelQD sequences with site-specific ^{15}N or ^{15}N/^{13}C labeling (10% isotope enrichment); except for ^{15}N/^{13}C doubly labeled residues, the ^{15}N-edited H8 spectral region is shown to the right; H8 of ^{15}N-G18 was too broad to be observed in the ^{15}N-edited spectrum. (B) Imino proton spectral region of ^{32Br}TelQD acquired in a 90% H_2O/10% D_2O buffer (top) and after transfer from a 100% D_2O buffer with a 20 minutes delay time. (C) Portions of a ^{1}H-^{13}C HSQC spectrum of ^{32Br}TelQD ^{15}N/^{13}C labeled at G26 and G31 (10% isotope enrichment) showing (top) H1'(ω_2)-C1'(ω_1) and (bottom) H8(ω_2)-C8(ω_1) correlations. Experiments were performed at 20 °C in 20 mM potassium phosphate buffer, pH 7, supplemented with 100 mM KCl.

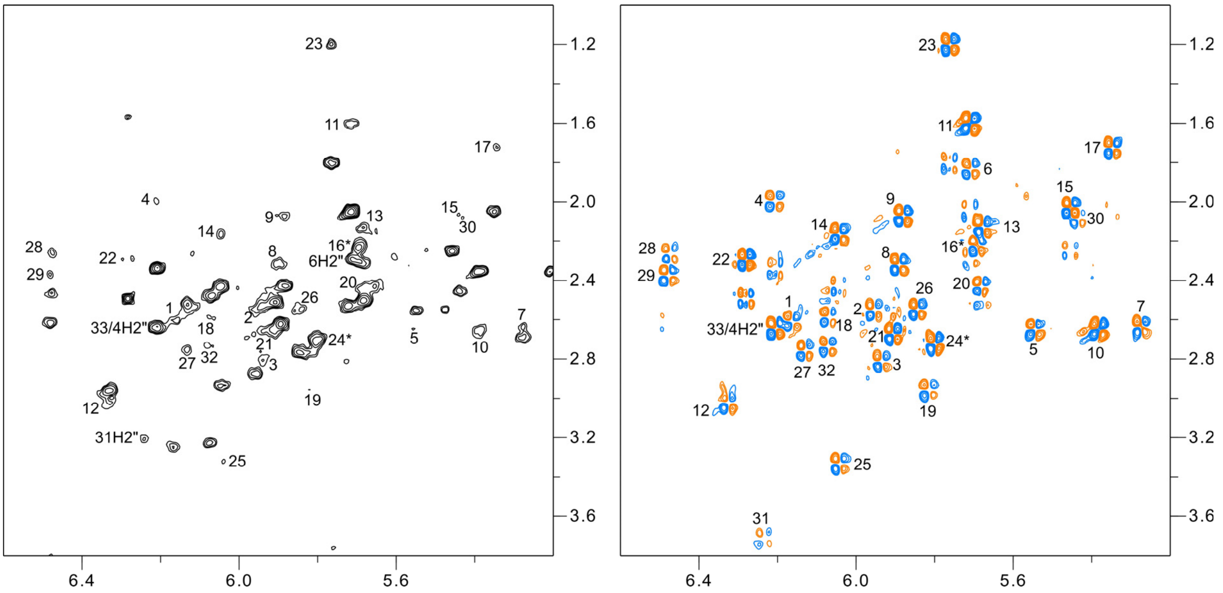

Figure S8. H1’(ω2)-H2’/H2”(ω1) spectral region of a NOESY spectrum (left, 80 ms mixing time) and of a DQF-COSY spectrum (right) for ^{32Br}TelQD. Only H1’-H2’ cross-peaks are labeled; starred labels for residues 16 and 24 indicate isochronous H2’/H2” resonances; signal overlap of residues 4 and 33 prevented their unambiguous sugar pucker assignment. Spectra were acquired at 20 °C in 20 mM potassium phosphate buffer (100% D_2O), pH 7.0, supplemented with 100 mM KCl.

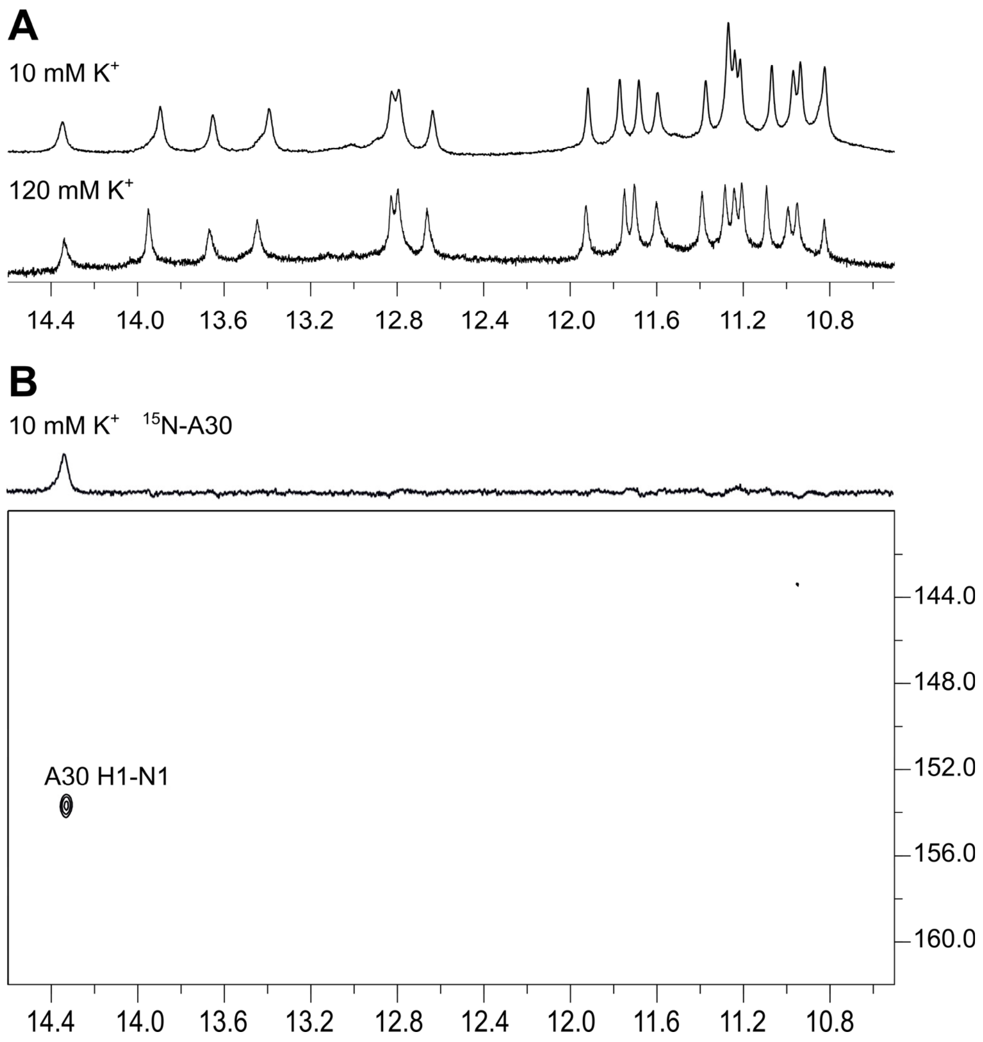

Figure S9. (A) Imino proton spectral region of ^{32Br}TelQD in a 10 mM and 120 mM K^+ buffer at 5 °C, demonstrating its essentially unaffected conformation. (B) Portion of a 2D ^{1}H-^{15}N HMQC spectrum of ^{32Br}TelQD with site-specifically ^{15}N-labeled A30 (10% ^{15}N enrichment) in a 10 mM K^+ buffer, pH 7, at 5 °C; a corresponding ^{15}N-edited 1D spectrum is shown on top.

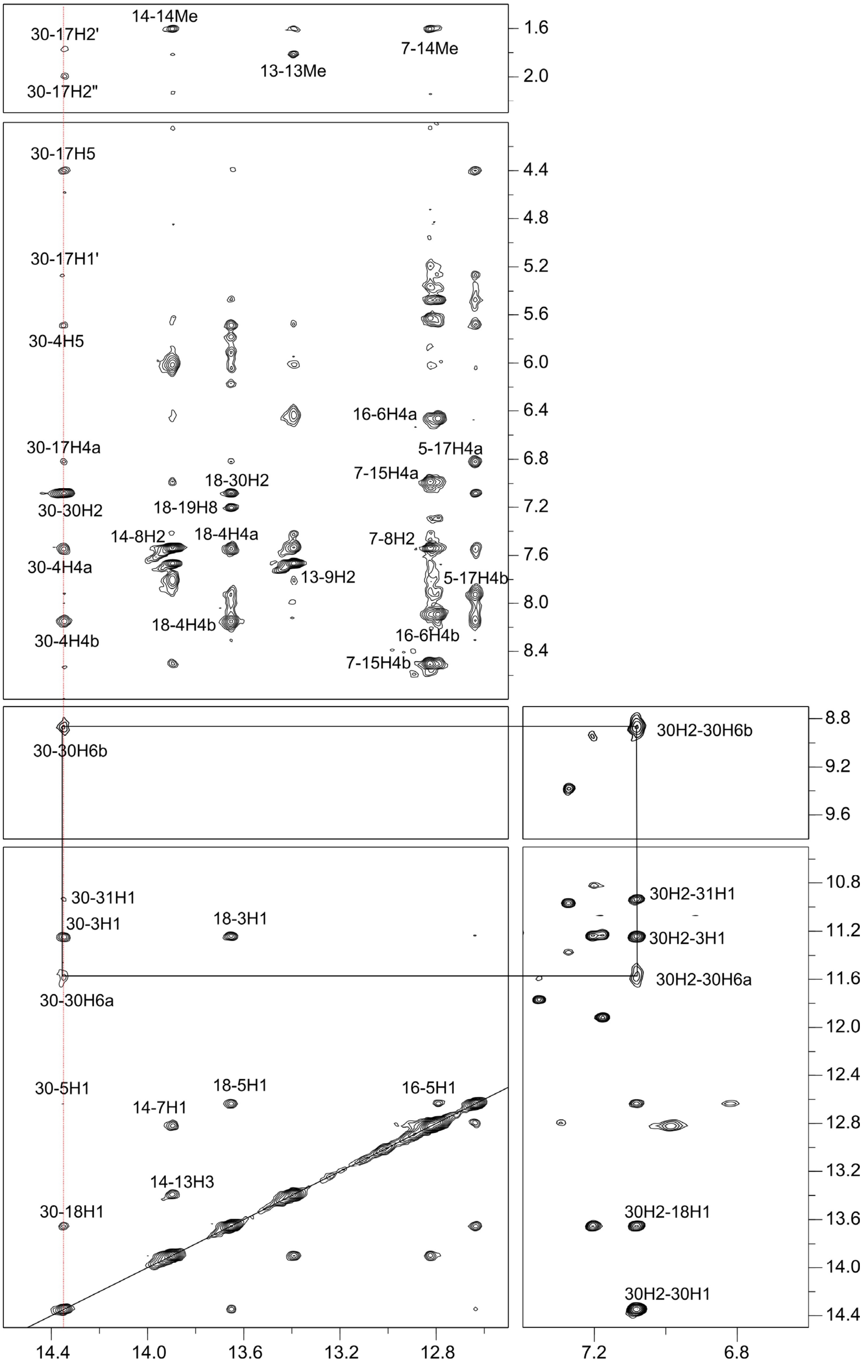

Figure S10. Regions of a NOESY spectrum (300 ms mixing time) for ^{32Br}TelQD at 5 °C; NOE contacts of the $AH^{+}30$ imino proton are indicated by the vertical red dotted line. The spectrum was acquired in 10 mM potassium phosphate buffer, pH 7.

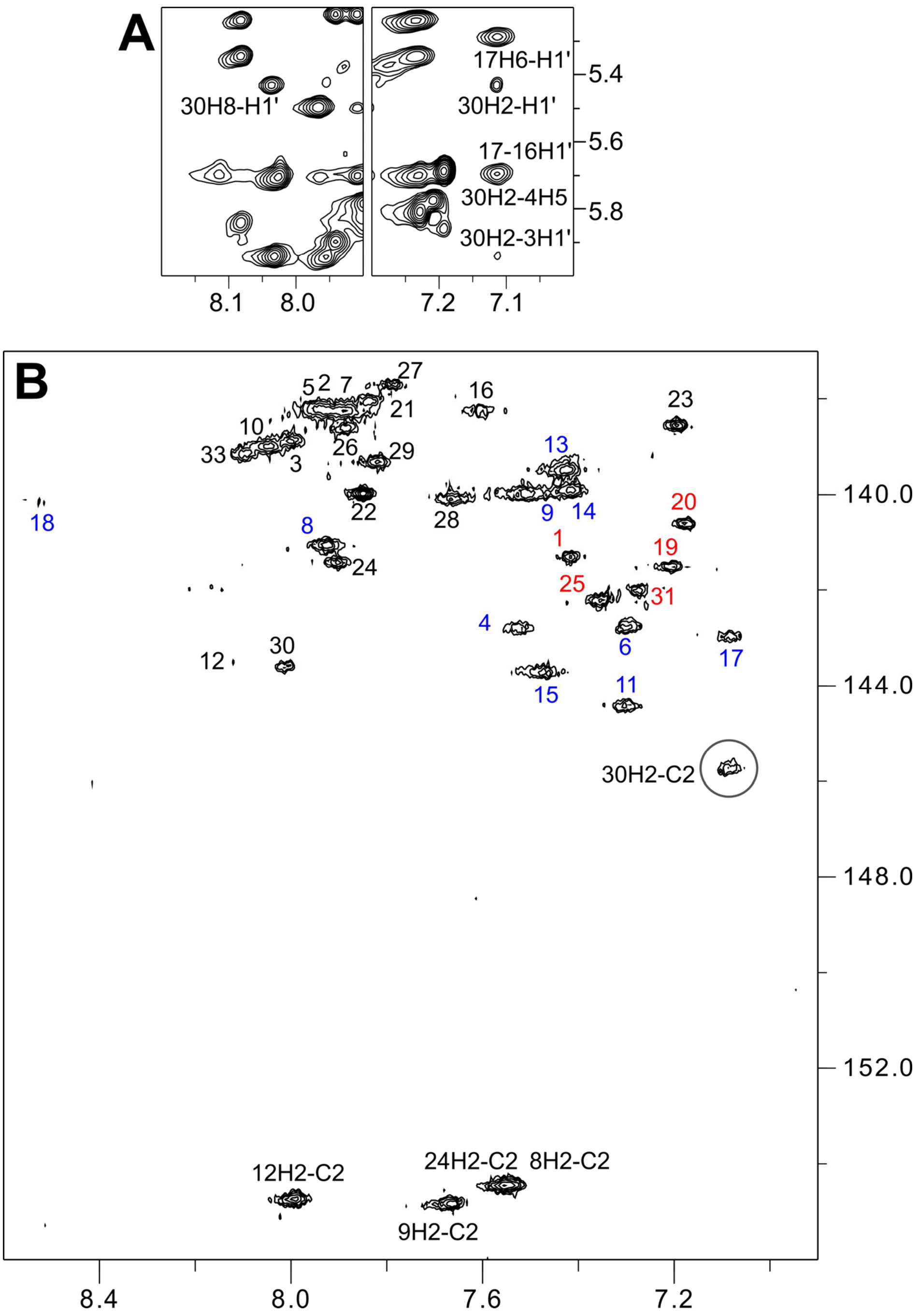

Figure S11. (A) NOESY spectral regions (300 ms mixing time) showing A30 H8/H2(ω2)-H1'(ω1) connectivities for assigning A30 H2. (B) ^{1}H-^{13}C HSQC spectrum of ^{32Br}TelQD showing H8/H6(ω2)-C8/C6(ω1) and adenine H2(ω2)-C2(ω1) correlations; *syn*-G residues of the G-core and residues of the hairpin duplex are labeled with red and blue color, respectively; the H2(ω2)-C2(ω1) correlation of protonated AH^{+}30 is highlighted by a circle. Spectra were acquired at 5 °C in 10 mM potassium phosphate buffer, pH 7.0, with either 100% D_2O (A) or 90% H_2O/10% D_2O (B).

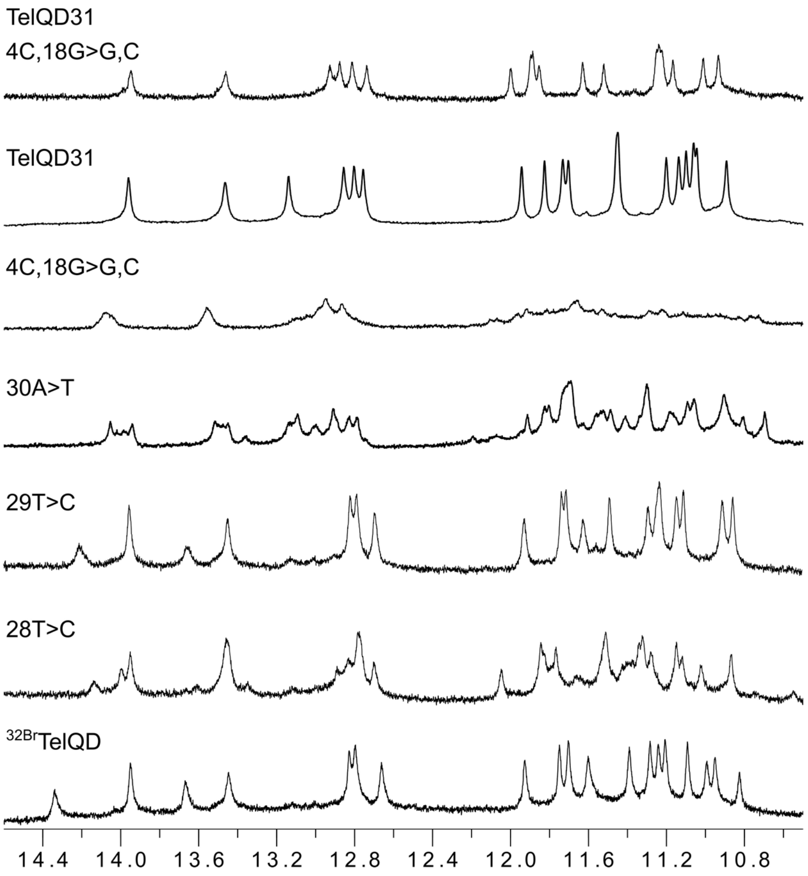

Figure S12. Imino proton spectral region of ^{32Br}TelQD mutants in a 120 mM K^+ buffer, pH 7. A spectrum of TelQD31 and of TelQD31 with a 4C,18G>G,C substitution for the interfacial base pair (top) shows conservation of the native (3+1) hybrid-2 fold in contrast to a corresponding mutant of antiparallel ^{32Br}TelQD. This demonstrates participation in additional interactions for the C4·G18 base pair in ^{32Br}TelQD. All spectra were acquired at 5 °C.

Table S4. ^{1}H and ^{13}C chemical shifts δ of ^{32Br}TelQD.[a]

δ / ppm	H8/H6	H1/H3	H1’	H2’/H2”	H3’	H5/H2/Me	C8/C6	C2
G1	7.42	11.68	6.13	2.58/3.21	5.01	-	141.35	-
G2	7.91	11.90	5.92	2.52/2.84	5.11	-	138.14	-
G3	7.95	11.24	5.90	2.78/2.50	5.01	-	138.76	-
C4	7.54	-	6.17	1.96/2.61	5.02	5.70	142.96	-
G5	7.91	12.64	5.52	2.61/2.52	4.95	-	138.13	-
C6	7.28	-	5.68	1.80/2.26	4.81	5.45	142.70	-
G7	7.85	12.77	5.24	2.61/2.65	4.92	-	138.19	-
A8	7.89	-	5.86	2.29/2.59	4.94	7.55	141.03	154.45
A9	7.44	-	5.85	2.04/2.39	4.86	7.67	139.83	154.96
G10	8.04	n.d.[b]	5.35	2.62/2.32	4.80	-	138.96	-
C11	7.17	-	5.68	1.58/2.02	4.40	5.18	143.44	-
A12	8.06	-	6.29	3.00/2.93	4.80	8.02	143.16	155.44
T13	7.39	13.32	5.65	2.10/2.47	4.75	1.82	139.39	-
T14	7.39	13.88	6.01	2.12/2.39	4.84	1.60	139.84	-
C15	7.45	-	5.42	2.00/2.22	4.74	5.63	143.69	-
G16	7.58	12.77	5.66	2.20/2.20	4.70	-	138.17	-
C17	7.04	-	5.32	1.69/2.02	4.56	4.43	142.79	-
G18	8.47	13.62	6.04	2.55/3.19	4.98	-	140.20[c]	-
G19	7.18	11.23	5.79	2.92/2.74	4.92	-	141.44	-
G20	7.18	10.83	5.65	2.39/2.28	4.92	-	140.64	-
G21	7.84	11.17	5.88	2.63/2.49	5.09	-	138.05	-
T22	7.82	n.d.[b]	6.25	2.27/2.47	4.84	1.95	140.02	-
T23	7.18	n.d.[b]	5.73	1.17/1.77	4.59	1.55	138.62	-
A24	7.85	-	5.77	2.67/2.68	4.92	7.51	141.13	155.32
G25	7.34	11.04	6.01	3.30/2.90	4.87	-	142.16	-
G26	7.86	11.58	5.81	2.51/2.73	5.04	-	138.58	-
G27	7.83	11.00	6.11	2.72/2.49	4.93	-	137.79	-
T28	7.65	n.d.[b]	6.44	2.34/2.58	4.95	1.70	140.24	-
T29	7.81	n.d.[b]	6.44	2.23/2.44	4.80	2.01	139.42	-
$AH^{+}30$	8.02	14.31	5.40	2.04/2.43	4.79	7.13	143.60[c]	145.75[c]
G31	7.26	10.92	6.20	3.69/3.17	4.95	-	142.01[c]	-
G32	-	11.36	6.03	2.69/2.44	5.07	-	n.d.[b]	-
G33	8.06	11.69	6.17	2.61/2.31	4.69	-	139.16	-

[a]At 20 ºC in 20 mM potassium phosphate buffer (90% H_2O/10% D_2O), pH 7, supplemented with 100 mM KCl. [b]n.d. = not determined. [c]At 5 ºC in 10 mM potassium phosphate buffer, pH 7.

Table S5. NMR restraints and structural statistics of ^{32Br}TelQD.

structure	^{32Br}TelQD
NOE distance restraints	
intra-residual	139
exchangeable	1
inter-residual	268
exchangeable	115
other restraints:	
hydrogen bonds	80
dihedral angles	61
planarity	9
chirality	155
structural statistics:	
pairwise heavy atom RMSD value (Å)	
all residues	1.49 ± 0.36
G-tetrad core	0.49 ± 0.09
NOE violations:	
number of NOE violations > 0.2 Å	0
maximum violation (Å)	0.188
mean NOE violation (Å)	0.004 ± 0.0005
deviations from idealized geometry:	
bond lengths (Å)	0.01 ± 0.0001
bond angles (degree)	2.3 ± 0.03

Spectral assignment of the 1:1 TelQD - Phen-DC$_3$ complex

With unambiguous resonance assignments by the use of site-specifically ^{15}N-enriched oligonucleotides, starting points for G-column assignments could be established (Figure S13). Having identified additional strong cytosine H6-H5 NOE cross-peaks through their correlations in a DQF-COSY spectrum, five *syn*-guanosine residues could be assigned based on their strong intra-nucleotide H8-H1’ NOE cross-peaks (Figure S14B). The first G-column from G1 to G3 was found to comprise *syn-anti-anti* steps. In contrast to the free TelQD hybrid, no sequential connectivity could be traced from G3 to C4. However, continuous NOE walks could be followed from C4 to G18. The second G-column from G19 to G21 was found to comprise *syn-syn-anti* steps. Again, continuous NOE walks could be followed from G21 to A24. Finally, the third (G25 to G27) and the fourth G-column (G31 to G33) were found to each have *syn-anti-anti* steps. Notably, there is no NOE connectivity from G27 to the third loop. On the other hand, a long-range contact from A30 H8 to G25 H1’ could be observed (Figure S14B). Apparently, the fold of TelQD complexed with Phen-DC$_3$ resembles the fold of TelQD31, adopting a hybrid-2 topology with a -(llp) loop progression.

With some of the G-core imino protons already assigned by specific ^{15}N isotope labeling, imino proton assignments of the quadruplex domain were complemented by H1-H1 and H1-H8 NOE crosspeaks (Figure S15B,D). These confirmed the arrangement of G-columns and a mixed homopolar/heteropolar tetrad stacking. Thus, polarities of the three tetrads run along G1→G31→G25→G21, G2→G20→G26→G32, and G3→G19→G27→G33. Duplex guanine H1 and thymine H3 imino protons were assigned based on their strong NOE contacts to amino protons of base-paired cytosine and to H2 of base-paired adenine, respectively. Additionally, G18 H1 was unambiguously assigned through a corresponding ^{15}N-G18 labeled sample. Of note, the G18 H1 signal is rather upfield-shifted and resonates within the spectral region typical of the Hoogsteen hydrogen-bonded G imino protons. Such a shielding may result from the ligand intercalating at the QD junction.[11]

Resonances of the ligand were assigned based on DQF-COSY and NOESY experiments (Figures S14 and S15; for proton numbering of the ligand see below). Symmetry-related protons of the phenanthroline moiety are nearly isochronous. Phenanthroline protons could be easily identified and distinguished from those of the quinoline ring system by strong NOE cross-peaks observed between H1/H1’ and H2/H2’ and COSY cross-peaks correlating H2/H2’ and H3/H3’. The DQF-COSY spectrum shows two spin systems H7/H7’ to H10/H10’ for the two quinoline sidearms (Figure S15C). Isochronicity of some proton resonances within the quinoline ring

system, e.g., H7 and H8 or H9’ and H10’, hamper their assignments but these were aided by a better dispersion of cross-peaks along the ^{13}C dimension in a ^{1}H-^{13}C HSQC experiment. This allowed for the unambiguous identification of all quinoline protons (Figure S16). Amide protons NH4/NH4’ gave rise to two separate resonances, featuring strong NOE contacts with H11/H11’ of the quinoline moiety. On the other hand, N-methyl protons 6Me/6’Me could be assigned through their NOE contacts to various neighboring protons within the quinoline ring system (Figure S14A). Notably, the N-methyl protons were found to resonate close to the water resonance.

A total of 100 NOE contacts were observed between ligand and QD hybrid protons, the latter mostly being located within the 3’-tetrad (G3, G19, G27, and G33) as well as in the interfacial C4·G18 Watson-Crick base pair of the duplex stem-loop. Several contacts of phenanthroline protons fix the phenanthroline moiety between residues G3, G19, C4, and G18, thus being sandwiched within the QD junction. On the other hand, quinoline protons showed NOE contacts to G27 and G33 and indicate that the Phen-DC_3 sidearms are located above the 3’-tetrad, capping the open tetrad side at the QD interface. Finally, based on relative intensities of H1’-H2’ and H1’-H2” DQF-COSY cross-peaks, 28 out of 33 residues were found to adopt a south-type sugar pucker (Figure S17).

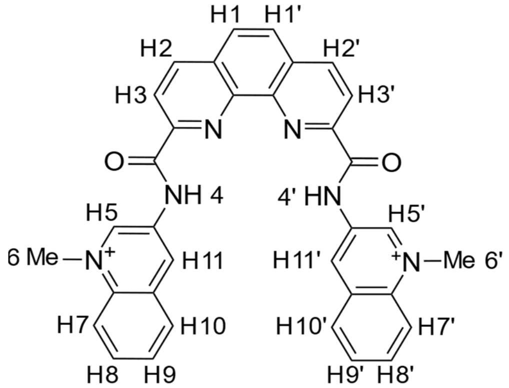

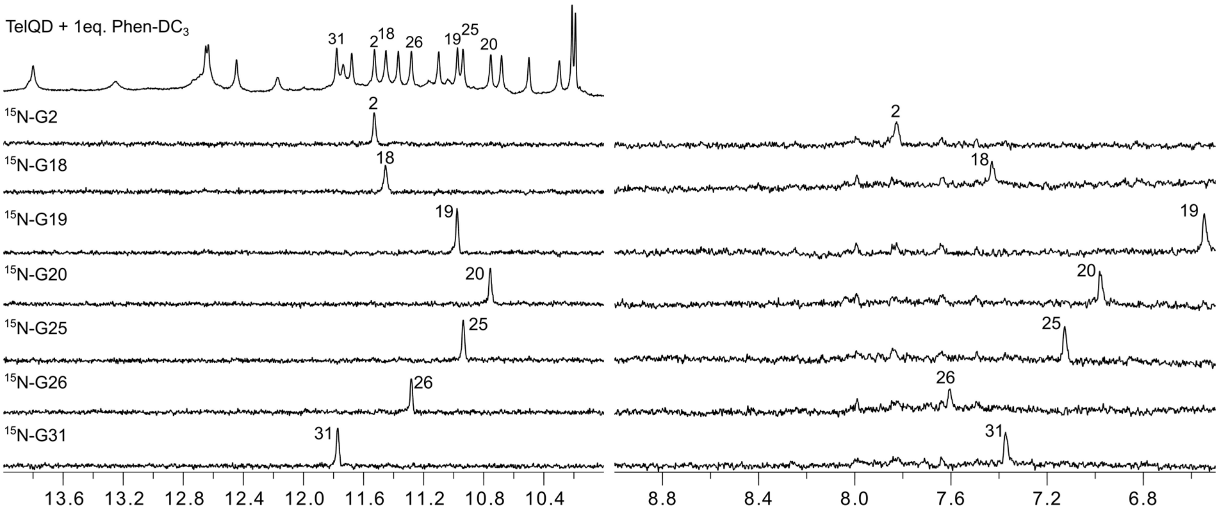

Figure S13. Imino proton spectral region of a 1:1 TelQD – Phen-DC3 complex (top) and ^{15}N-edited spectra for corresponding complexes with site-specifically ^{15}N labeled TelQD sequences (10% ^{15}N enrichment); the ^{15}N-edited H8 spectral region is shown to the right. Spectra were acquired at 25 °C in a 120 mM K^+ buffer, pH 7.

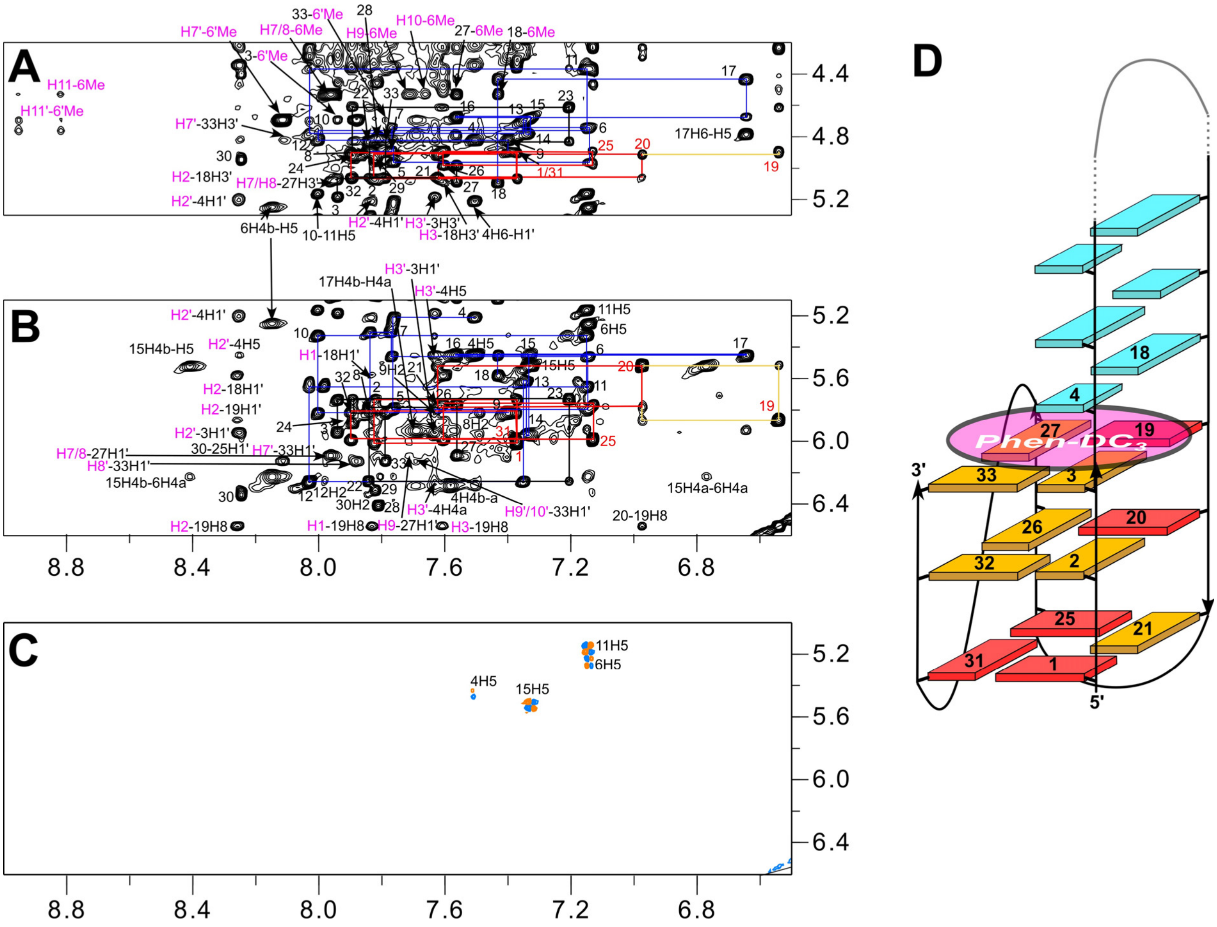

Figure S14. (A) H8/H6(ω_2)-H3'(ω_1) and (B) H8/H6(ω_2)-H1'(ω_1) NOESY spectral region of the 1:1 TelQD – Phen-DC_3 complex; NOE connectivities within the duplex domain are traced by blue lines; red lines forming a typical rectangular pattern connect two residues in a *syn-anti* step while yellow-colored lines connect the two *syn*-residues 19 and 20; *anti*- and *syn*-G residues are labeled in black and red color respectively; ligand protons are labeled in magenta. (C) DQF-COSY spectral region of the 1:1 TelQD – Phen-DC_3 complex showing cytosine H6-H5 cross-peaks. (D) Schematic structure of the 1:1 TelQD – Phen-DC_3 complex; *anti*- and *syn*-G residues of the G-core are colored orange and red whereas base pairs of the duplex stem-loop and the ligand are colored cyan and magenta, respectively. NOESY (300 ms mixing time) and DQF-COSY spectra were acquired at 25 °C in 120 mM K^+ buffer, pH 7.0.

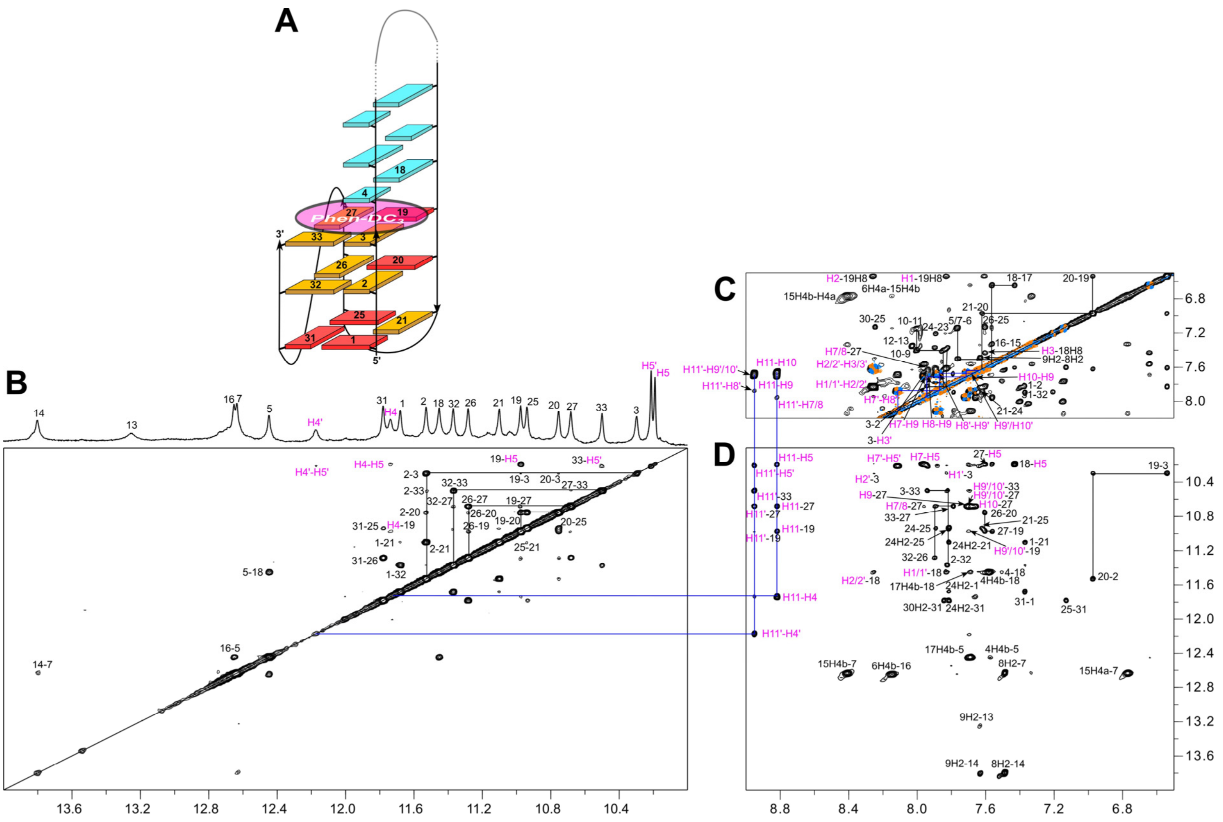

Figure S15. (A) Schematic structure of the 1:1 TelQD – Phen-DC3 complex; *anti*- and *syn*-G residues of the G-core are colored orange and red whereas base pairs of the duplex stem-loop and the ligand are colored cyan and magenta, respectively. (B) H1-H1 2D NOE spectral region of the complex and a corresponding 1D spectrum with imino resonance assignments shown on top. (C) Superposition of DQF-COSY and NOESY spectral regions showing correlations between aromatic protons; black lines trace NOE connectivities of DNA protons whereas blue lines follow connectivities for spin-coupled ligand protons. (D) H8/H6(ω_2)-H1(ω_1) NOESY spectral region. In (B-D), DNA and ligand protons are labeled in black and magenta, respectively. NOESY (300 ms mixing time) and DQF-COSY spectra were acquired at 25 °C in 120 mM K^+ buffer, pH 7.0.

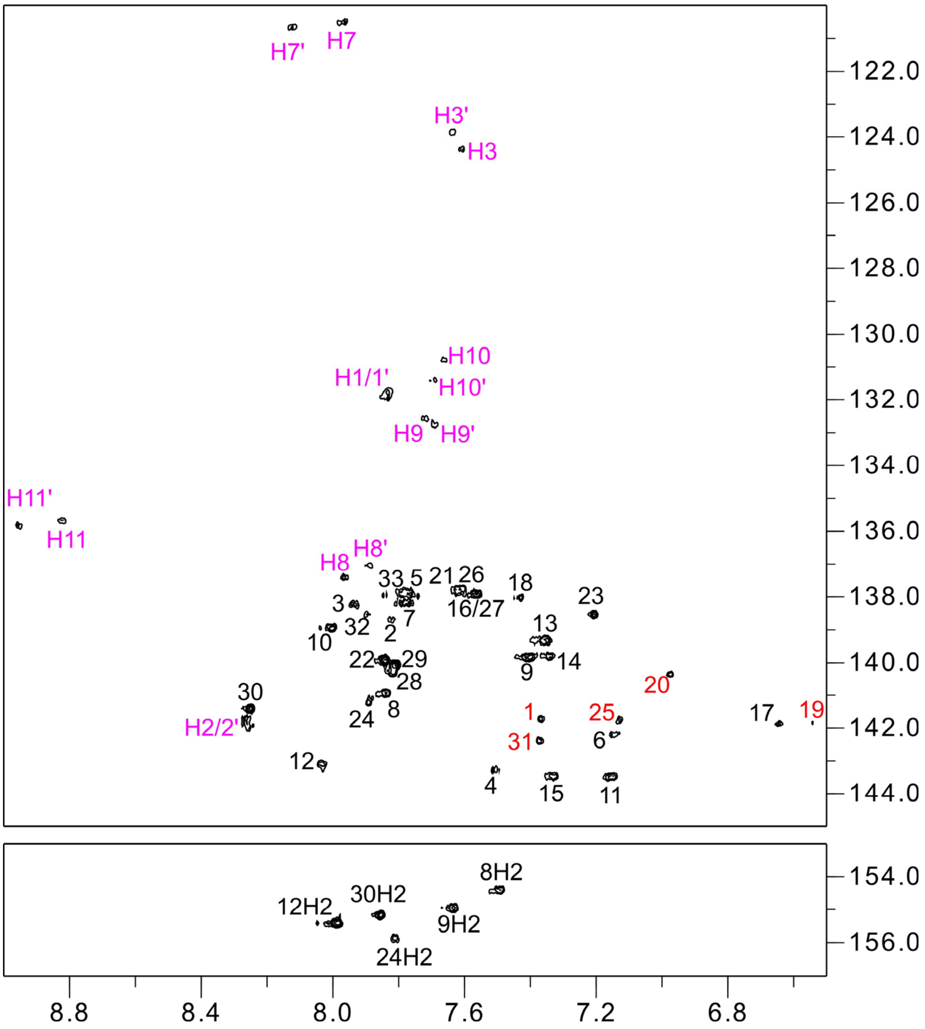

Figure S16. ^{1}H-^{13}C HSQC spectrum of a 1:1 TelQD – Phen-DC_3 complex showing H8/H6(ω_2)-C8/C6(ω_1) and additional correlations of aromatic ligand protons (top) as well as adenine H2(ω_2)-C2(ω_1) correlations (bottom); cross-peaks of *syn*-G residues and of the ligand are labeled in red and magenta, respectively. Spectra were acquired at 25 °C in a 120 mM K^+ buffer, pH 7.0.

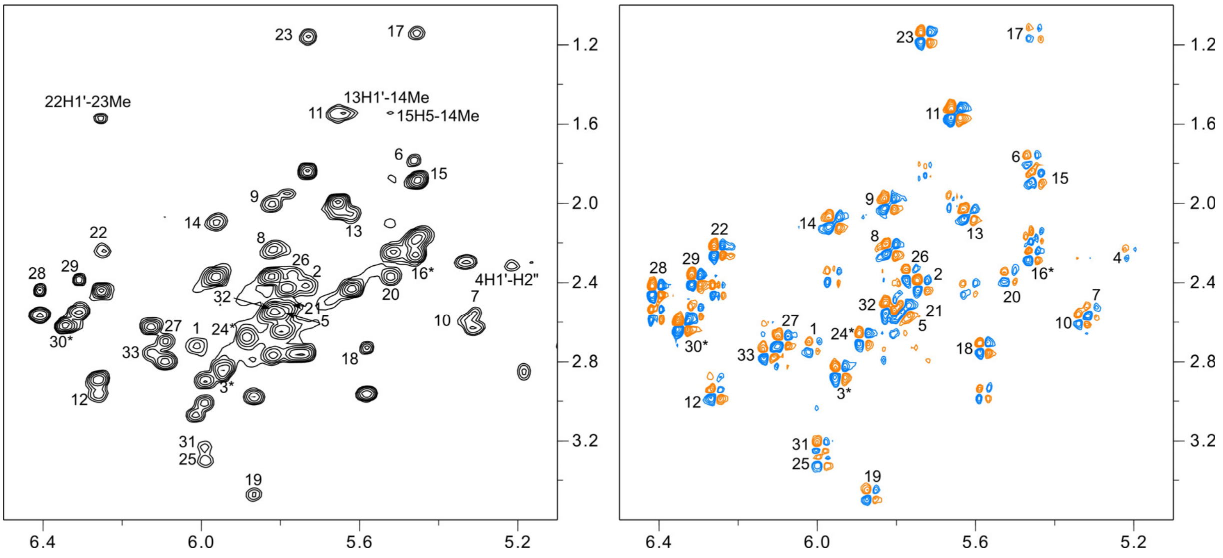

Figure S17. H1’(ω_2)-H2’/H2”(ω_1) spectral region of a NOESY spectrum (left, 80 ms mixing time) and of a DQF-COSY spectrum (right) for a 1:1 TelQD – Phen-DC$_3$ complex. Only H1’-H2’ cross-peaks are labeled; starred labels indicate overlapped cross-peaks due to nearly isochronous H2’/H2” resonances. Spectra were acquired at 25 °C in a 120 mM K^+ buffer (100% D_2O), pH 7.0.

Table S6. ^{1}H and ^{13}C chemical shifts δ of the 1:1 TelQD – Phen-DC$_3$ complex.[a]

δ / ppm	H8/H6	H1/H3	H1'	H2'/H2"	H3'	H5/H2/Me	C8/C6	C2
G1	7.37	11.68	6.01	2.73/3.07	4.91	-	141.73	-
G2	7.82	11.53	5.74	2.41/2.76	5.07	-	138.70	-
G3	7.94	10.30	5.94	2.85/2.84	5.19	-	138.23	-
C4	7.50	-	5.21	2.26/2.32	4.83	5.45	143.30	-
G5	7.76	12.45	5.80	2.55/2.64	4.97	-	137.92	-
C6	7.14	-	5.46	1.78/2.20	4.74	5.25	142.21	-
G7	7.77	12.64	5.31	2.56/2.63	4.89	-	138.18	-
A8	7.84	-	5.82	2.23/2.55	4.90	7.49	140.94	154.42
A9	7.40	-	5.82	2.00/2.37	4.82	7.63	139.85	154.96
G10	8.00	n.d.[b]	5.33	2.59/2.30	4.75	-	138.95	-
C11	7.15	-	5.65	1.55/1.99	4.37	5.17	143.47	-
A12	8.03	-	6.26	2.96/2.89	4.79	7.98	143.11	155.42
T13	7.35	13.25	5.62	2.05/2.43	4.70	1.78	139.33	-
T14	7.34	13.80	5.96	2.09/2.38	4.79	1.55	139.80	-
C15	7.33	-	5.45	1.87/2.16	4.68	5.52	143.45	-
G16	7.56	12.65	5.46	2.26/2.26	4.68	-	137.93	-
C17	6.64	-	5.46	1.15/1.89	4.43	4.79	141.85	-
G18	7.43	11.45	5.58	2.73/2.96	5.09	-	138.02	-
G19	6.54	10.98	5.87	3.47/2.98	4.90	-	141.87	-
G20	6.97	10.76	5.52	2.37/2.25	4.91	-	140.37	-
G21	7.62	11.10	5.78	2.53/2.43	5.06	-	137.80	-
T22	7.84	n.d.[b]	6.25	2.24/2.44	4.83	1.95	139.89	-
T23	7.21	n.d.[b]	5.73	1.16/1.84	4.61	1.57	138.53	-
A24	7.89	-	5.89	2.68/2.68	4.92	7.81	141.20	155.89
G25	7.13	10.94	5.99	3.30/2.89	4.90	-	141.78	-
G26	7.60	11.28	5.77	2.36/2.76	4.98	-	137.83	-
G27	7.56	10.68	6.09	2.70/2.80	5.09	-	137.92	-
T28	7.81	n.d.[b]	6.41	2.44/2.57	4.81	1.99	140.29	-
T29	7.82	n.d.[b]	6.31	2.39/2.55	4.94	2.07	140.05	-
A30	8.25	-	6.34	2.62/2.62	4.95	7.85	141.37	155.18
G31	7.37	11.78	5.99	3.23/3.01	4.90	-	142.38	-
G32	7.90	11.37	5.82	2.52/2.77	5.07	-	138.51	-
G33	7.79	10.50	6.13	2.75/2.63	4.82	-	137.90	-

[a]At 25 ºC in 20 mM potassium phosphate buffer (90% H_2O/10% D_2O), pH 7, supplemented with 100 mM KCl.
[b]n.d. = not determined.

Table S7. ^{1}H chemical shifts *δ* of bound PhenDC3 in the 1:1 complex with TelQD.[a]

δ / ppm	H1	H2	H3	NH4	H5	6Me	H7	H8	H9	H10	H11
	7.83	8.26	7.61	11.74	10.19	4.53	7.96	7.96	7.71	7.66	8.82
δ / ppm	H1'	H2'	H3'	NH4'	H5'	6'Me	H7'	H8'	H9'	H10'	H11'
	7.83	8.25	7.63	12.17	10.21	4.70	8.11	7.88	7.69	7.69	8.95

[a]At 25 ºC in 20 mM potassium phosphate buffer (90% H_2O/10% D_2O), pH 7, supplemented with 100 mM KCl.

Table S8. Intermolecular NOE contacts observed between Phen-DC3 and TelQD protons.

very strong	strong	weak	very weak	ambiguous

	C4								G18					
	H6	H5	H4a	H4b	H1’	H2’	H2”	H3’	H1	H8	H1’	H2’	H2”	H3’
H1									weak		very weak			
H2									very weak		strong	weak	weak	very weak
H3										very weak	strong		strong	strong
NH4														
H5										very strong	weak	strong	strong	
H6(Me)										strong				
H7														
H8														
H9														
H10														
H11												very weak		
H1’					weak				ambiguous					
H2’	very weak	weak			weak	very weak	very weak		weak					
H3’		strong	strong											
NH4’														
H5’		weak	weak											
H6’(Me)														
H7’														
H8’														
H9’														
H10’														
H11’		weak	weak											

Table S8 continued.

	G3						G19						G27						G33					
	H1	H8	H1’	H2’	H2”	H3’	H1	H8	H1’	H2’	H2”	H3’	H1	H8	H1’	H2’	H2”	H3’	H1	H8	H1’	H2’	H2”	H3’
H1																								
H2																								
H3																								
NH4																								
H5																								
H6																								
H7																								
H8																								
H9																								
H10																								
H11																								
H1’																								
H2’																								
H3’																								
NH4’																								
H5’																								
H6’																								
H7’																								
H8’																								
H9’																								
H10’																								
H11’																								

Table S9. NMR restraints and structural statistics of the 1:1 TelQD - Phen-DC_3 complex.

structure	TelQD - Phen-DC_3 complex
NOE distance restraints	
intra-residual	145
inter-residual	269
exchangeable	101
intra-molecular (ligand)	2
ligand-DNA	100
other restraints:	
hydrogen bonds	80
dihedral angles	61
planarity	9
chirality	165
structural statistics:	
pairwise heavy atom RMSD value (Å)	
all residues	1.61 ± 0.34
G-tetrad core	0.60 ± 0.21
QD interface with bound ligand	0.39 ± 0.12
NOE violations:	
number of NOE violations > 0.2 Å	0.3 ± 0.5
maximum violation (Å)	0.236
mean NOE violation (Å)	0.003 ± 0.0006
deviations from idealized geometry:	
bond lengths (Å)	0.01 ± 0.0001
bond angles (degree)	2.3 ± 0.03

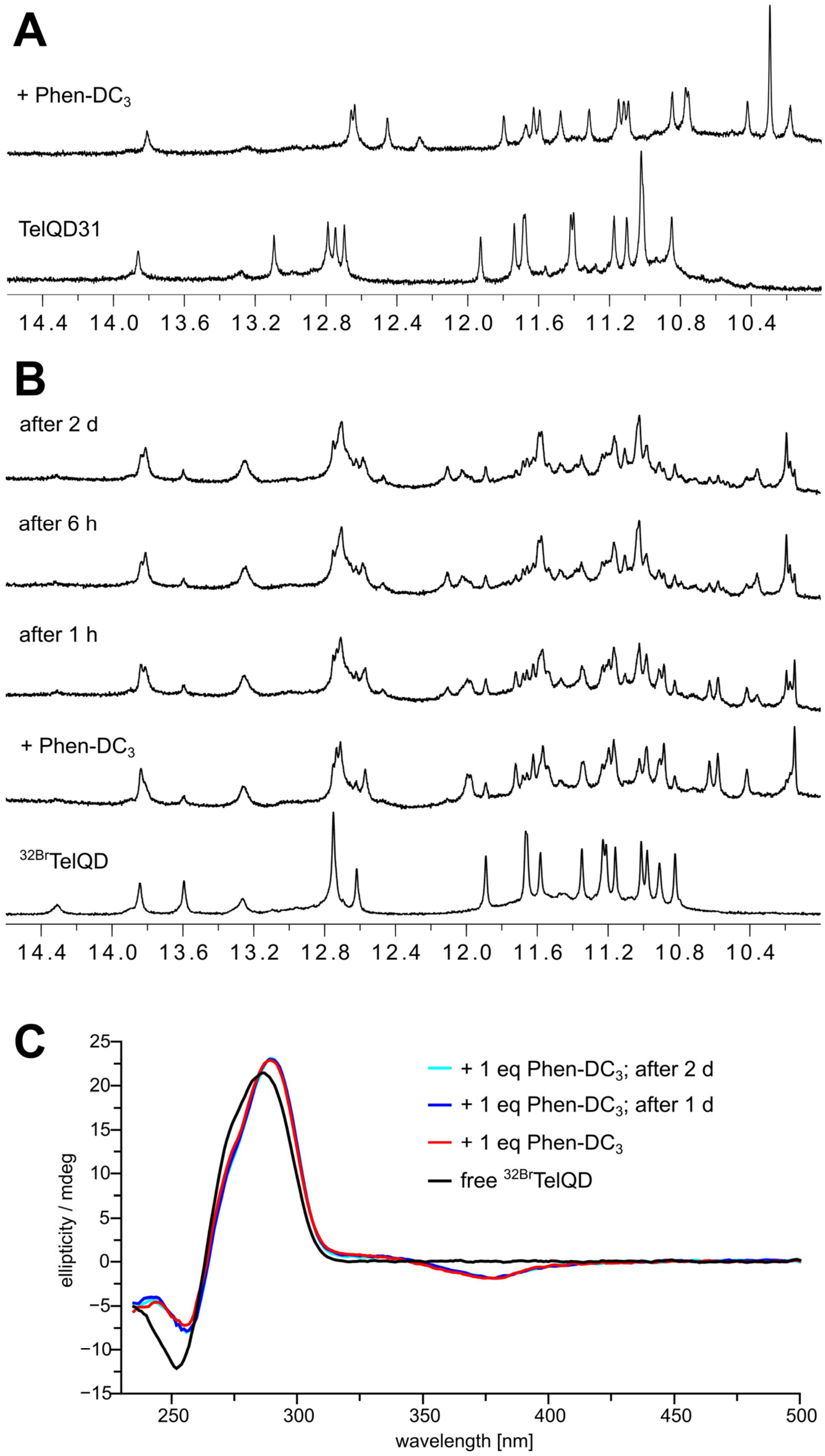

Figure S18. (A) Imino proton NMR spectral region of TelQD31 before (bottom) and immediately after addition of 1 eq. Phen-DC$_3$ (top). (B) Imino proton NMR spectral region of ^{32Br}TelQD before (bottom) and after addition of 1 eq. Phen-DC$_3$ at different time delays. (C) CD spectra of free and Phen-DC$_3$ bound ^{32Br}TelQD acquired immediately or 1-2 days after ligand addition. All experiments were performed at 25 °C with a 120 mM K^+ buffer, pH 7.0.

Spectral assignment of the 1:1 TelQD31 - Phen-DC_3 complex

NOE connectivity patterns of TelQD31 complexed with Phen-DC_3 show similarities to both the free TelQD31 and the TelQD – Phen-DC_3 complex, especially along the duplex domain (Figure S19B). Making use of a site-specific ^{15}N-labeling methodology, the first G-column comprising a *syn*-G1, *anti*-G2, and *anti*-G3 could be unambiguously identified (Figures S19 and S20). A similar approach could be applied for the second G-column *syn*-G19, *syn*-G20, and *anti*-G21. The third and the fourth G-columns were discriminated following unambiguous resonance assignments through ^{15}N-labeled G29 and G30 residues. Both of these G-columns feature *syn-anti-anti* steps. An interruption of continuous NOE connectivities between G3 and C4 suggests intercalation of Phen-DC_3 at the QD junction. The presence of five *syn*-guanosines is confirmed by their downfield-shifted C8 resonances in a ^{1}H-^{13}C HSQC experiment (Figure S21). Taken together, a hybrid-2 topology seems to have been conserved in the complex.

H8-H1 NOE contacts indicate a mixed homopolar/heteropolar tetrad stacking. Confirmed by unambiguous resonance assignments and additionally supported by H1-H1 NOE contacts, H8-H1 cross-peaks identified the location of adjacent G-columns and tetrad polarities with intra-tetrad hydrogen bonds running along G1→G29→G25→G21, G2→G20→G26→G30, and G3→G19→G27→G31 (Figure S19). A similar strategy as used before was employed for assigning duplex imino protons. Here, a significantly upfield-shifted G18 H1 resonance was again observed and suggests a corresponding Phen-DC_3 intercalation as already found for the TelQD complex. This was also confirmed by following the change of imino and H8 protons upon Phen-DC_3 binding to TelQD31. In a chemical shift footprint, most significant shifts are experienced by the upper tetrad and the first C·G Watson-Crick base pair of the duplex domain constituting the QD interface (Figure S22).

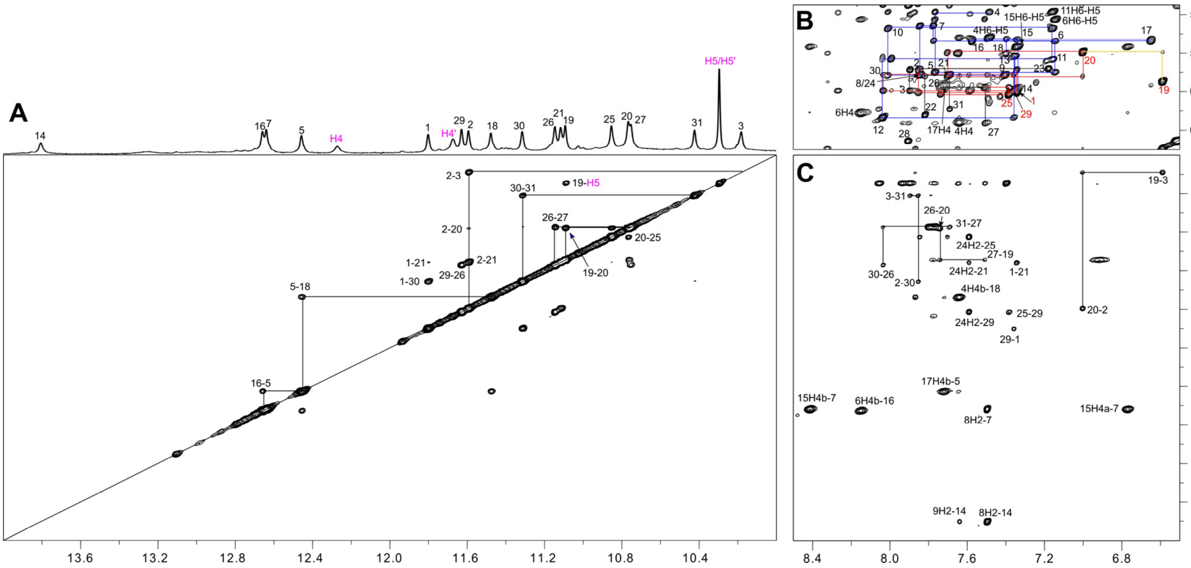

Figure S19. (A) H1-H1 2D NOE spectral region of TelQD31 complexed with Phen-DC_3 (1:1 molar ratio) and a corresponding 1D spectrum with imino resonance assignments shown on top; ligand protons are labeled in magenta. (B) H8/H6(ω_2)-H1'(ω_1) and (C) H8/H6(ω_2)-H1(ω_1) NOESY spectral region; in (B), NOE connectivities within the duplex domain are traced by blue lines; red lines forming a typical rectangular pattern connect two residues in a *syn-anti* step while yellow-colored lines connect the two residues in a *syn-syn* step; *anti-* and *syn-*G residues are labeled in black and red color, respectively. NOESY spectra (300 ms mixing time) were acquired at 25 °C in 120 mM K^+ buffer, pH 7.0.

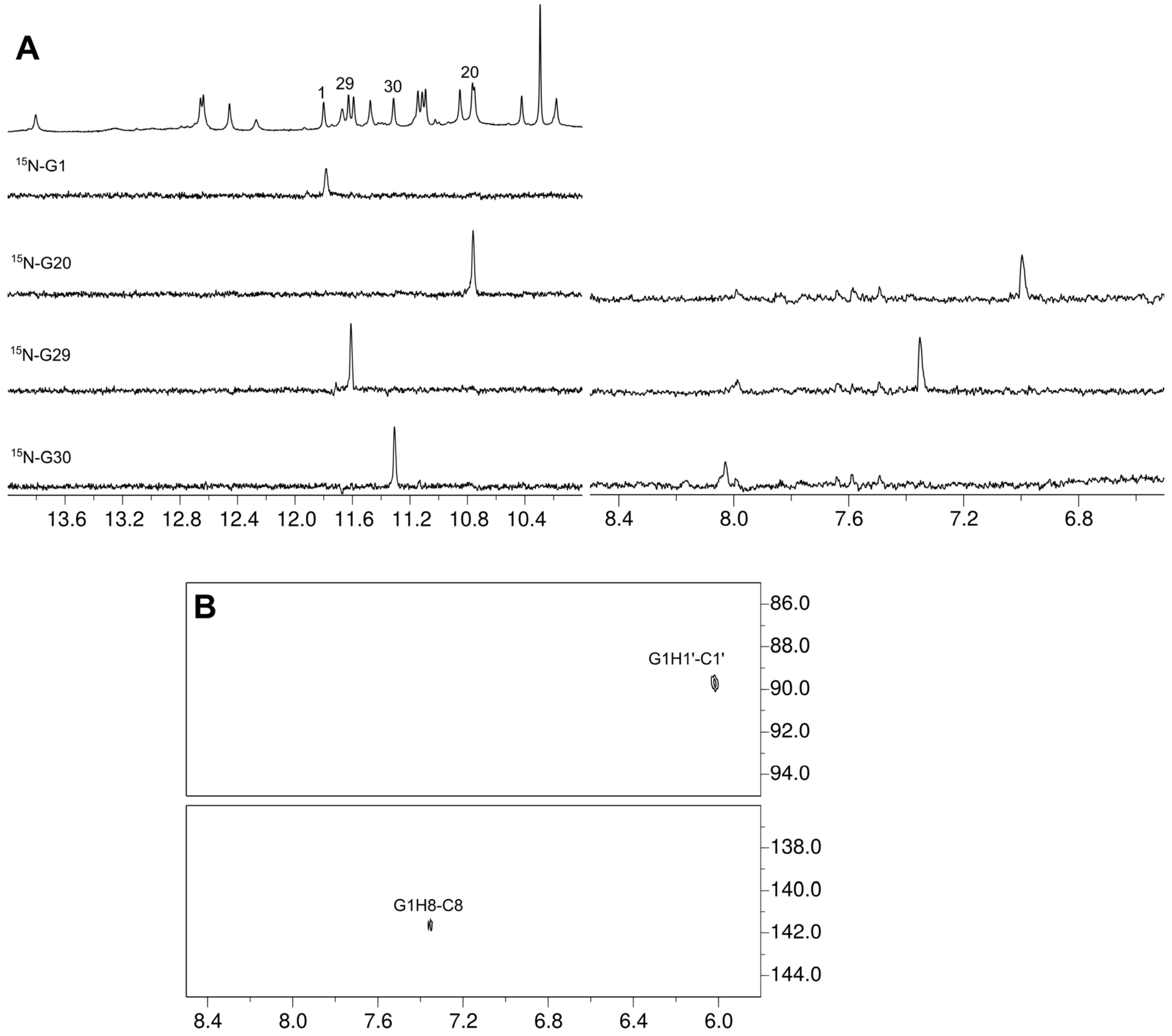

Figure S20. (A) Guanine imino proton spectral region of non-labeled TelQD31 complexed with Phen-DC3 (1:1 molar ratio, top) and corresponding ^{15}N-edited spectra using TelQD31 sequences with site-specific ^{15}N labeling (10% isotope enrichment); except for G1, the ^{15}N-edited H8 spectral region is shown to the right. (B) Portions of a ^{1}H-^{13}C HSQC spectrum of ligand-bound TelQD31 ^{13}C labeled at G1 (10% isotope enrichment) showing (top) H1'(ω_2)-C1'(ω_1) and (bottom) H8(ω_2)-C8(ω_1) correlations. Experiments were performed at 25 °C in 120 mM K^+ buffer, pH 7.

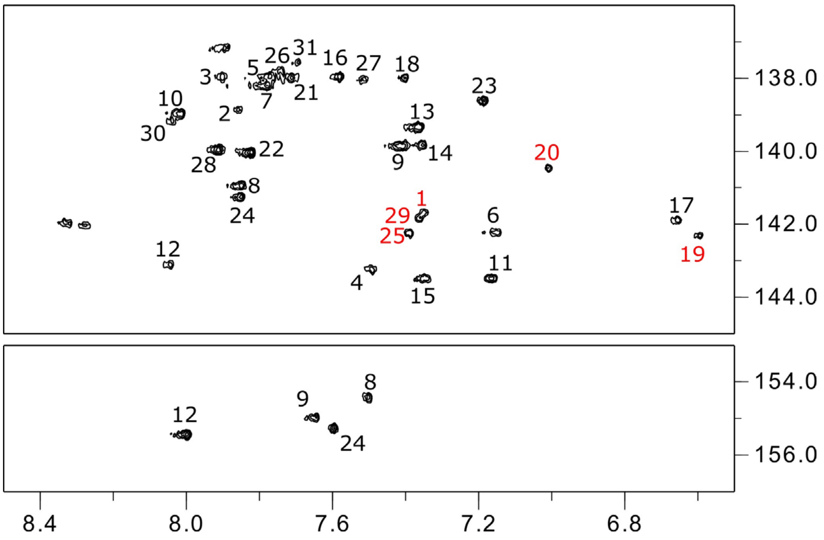

Figure S21. ^{1}H-^{13}C HSQC spectrum of TelQD31 complexed with Phen-DC3 (1:1 molar ratio) showing the H6/H8(ω2)-C6/C8(ω1) (top) and the adenine H2(ω2)-C2(ω1) spectral region (bottom); *syn*-G residues are labeled in red.

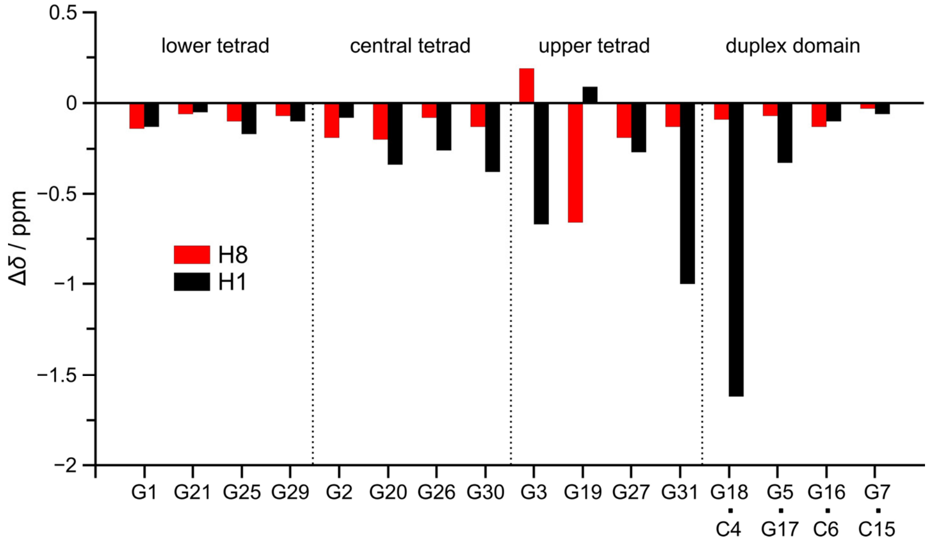

Figure S22. Residue-dependent chemical shift perturbation Δδ of H1 and H8 protons upon complex formation of TelQD31 with Phen-DC3 at 25 °C in 120 mM K^+, pH 7.0.

Table S10. ^{1}H and ^{13}C chemical shifts δ of a 1:1 TelQD31 – Phen-DC$_3$ complex.[a]

δ / ppm	H8/H6	H1/H3	H1'	H2'/H2''[c]	H5/H2/Me	C8/C6	C2
G1	7.35	11.80	6.01	2.69/3.05	-	141.71	-
G2	7.85	11.59	5.77	2.48/2.76	-	138.85	-
G3	7.90	10.18	5.99	2.85/2.85	-	137.97	-
C4	7.49	-	5.17	2.22/2.31	5.44	143.24	-
G5	7.77	12.46	5.80	2.56/2.64	-	137.94	-
C6	7.15	-	5.47	1.79/2.20	5.25	142.24	-
G7	7.77	12.64	5.32	2.55/2.64	-	138.18	-
A8	7.84	-	5.83	2.24/2.55	7.50	140.95	154.44
A9	7.41	-	5.83	2.01/2.38	7.65	139.84	154.97
G10	8.01	n.d.[b]	5.34	2.31/2.59	-	138.96	-
C11	7.16	-	5.66	1.55/2.01	5.17	143.50	-
A12	8.04	-	6.27	2.90/2.97	8.00	143.13	155.42
T13	7.36	n.d.[b]	5.63	2.06/2.44	1.79	139.33	-
T14	7.35	13.81	5.98	2.10/2.38	1.56	139.83	-
C15	7.34	-	5.46	1.88/2.18	5.53	143.49	-
G16	7.58	12.65	5.47	2.28/2.28	-	137.95	-
C17	6.65	-	5.46	1.13/1.89	4.80	141.90	-
G18	7.40	11.48	5.60	2.72/2.99	-	137.99	-
G19	6.59	11.09	5.90	2.93/3.41	-	142.33	-
G20	7.00	10.76	5.59	2.40/2.49	-	140.44	-
G21	7.70	11.12	5.84	2.42/2.56	-	137.97	-
T22	7.82	n.d.[b]	6.24	2.24/2.46	1.94	140.04	-
T23	7.18	n.d.[b]	5.76	1.19/1.69	1.54	138.61	-
A24	7.85	-	5.83	2.66/2.68	7.59	141.26	155.29
G25	7.39	10.85	6.03	2.92/3.36	-	142.25	-
G26	7.74	11.14	5.96	2.46/2.88	-	137.82	-
G27	7.51	10.75	6.33	2.87/2.91	-	138.02	-
T28	7.91	n.d.[b]	6.51	2.42/2.75	2.02	139.96	-
G29	7.36	11.63	5.99	2.93/3.04	-	141.84	-
G30	8.04	11.31	5.82	2.61/2.72	-	139.15	-
G31	7.69	10.42	6.18	2.67/2.76	-	137.56	-

[a]At 25 ºC in 20 mM potassium phosphate buffer (90% H_2O/10% D_2O), pH 7, supplemented with 100 mM KCl. [b]n.d. = not determined. [c]No stereospecific assignment was done for these protons.

References

[1] W. F. Vranken, W. Boucher, T. J. Stevens, R. H. Fogh, A. Pajon, M. Llinas, E. L. Ulrich, J. L. Markley, J. Ionides, E. D. Laue, *Proteins Struct. Funct. Bioinforma.* **2005**, *59*, 687–696.

[2] A. T. Phan, D. J. Patel, *J. Am. Chem. Soc.* **2002**, *124*, 1160–1161.

[3] C. D. Schwieters, J. J. Kuszewski, G. Marius Clore, *Prog. Nucl. Magn. Reson. Spectrosc.* **2006**, *48*, 47–62.

[4] M. Zgarbová, J. Šponer, M. Otyepka, T. E. Cheatham, R. Galindo-Murillo, P. Jurečka, *J. Chem. Theory Comput.* **2015**, *11*, 5723–5736.

[5] E. Vanquelef, S. Simon, G. Marquant, E. Garcia, G. Klimerak, J. C. Delepine, P. Cieplak, F.-Y. Dupradeau, *Nucleic Acids Res.* **2011**, *39*, W511-W517.

[6] W. L. Jorgensen, J. Chandrasekhar, J. D. Madura, R. W. Impey, M. L. Klein, *J. Chem. Phys.* **1983**, *79*, 926–935.

[7] Y. M. Vianney, N. Schröder, J. Jana, G. Chojetzki, K. Weisz, *J. Am. Chem. Soc.* **2023**, *145*, 22194−22205.

[8] W. Humphrey, A. Dalke, K. Schulten, *J. Mol. Graph.* **1996**, *14*, 33-38.

[9] W. L. DeLano, *CCP4 Newsl. Protein Crystallogr.* **2002**, *40*, 82-92.

[10] K. W. Lim, A. T. Phan, *Angew. Chemie Int. Ed.* **2013**, *52*, 8566–8569.

[11] Y. M. Vianney, K. Weisz, *Nucleic Acids Res.* **2022**, *50*, 11948–11964.
